# Barriers and facilitators for strengthening primary health systems for person-centred multimorbid care in low-income and middle-income countries: a scoping review

**DOI:** 10.1136/bmjopen-2024-087451

**Published:** 2024-11-27

**Authors:** David Zezai, André Janse van Rensburg, Gbotemi Bukola Babatunde, Tasneem Kathree, Ruth Cornick, Naomi Levitt, Lara R Fairall, Inge Petersen

**Affiliations:** 1 Centre for Rural Health, University of KwaZulu-Natal College of Health Sciences, Durban, KwaZulu Natal, South Africa; 2 School of Public Health, University of the Western Cape Faculty of Community and Health Sciences, Cape Town, Western Cape, South Africa; 3 Department of Psychology, University of Denver, Graduate School of Professional Psychology, Denver, Colorado, USA; 4 Observatory, Department of Medicine, University of Cape Town, Cape Town, Western Cape, South Africa; 5 Knowledge Translation Unit, University of Cape Town, Cape Town, Western Cape, South Africa; 6 Division of Endocrinology, Department of Medicine, University of Cape Town, Cape Town, Western Cape, South Africa; 7 Chronic Disease Initiative for Africa, Department of Medicine, University of Cape Town, Cape Town, Western Cape, South Africa; 8 Global Health Institute, School of Life Course and Population Sciences, King's College London Faculty of Life Sciences & Medicine, London, UK; 9 Global Health Institute, King’s College London, London, UK

**Keywords:** Health Services, Chronic Disease, Primary Health Care, Multimorbidity, Person-Centered Care

## Abstract

**Abstract:**

**Objective:**

To understand barriers and facilitators for strengthening health systems for person-centred care of people with multiple long-term conditions-multimorbidity (MLTC-M) at the primary healthcare (PHC) level in low-income and middle-income countries (LMICs).

**Design:**

A scoping review.

**Methods:**

We adopted a systematic scoping review approach to chart literature guided by Arksey and O'Malley’s methodological framework. The review focused on studies conducted in LMICs’ PHC settings from January 2010 to December 2023. Papers were extracted from the following databases: PubMed, EBSCOhost and Google Scholar. Framework analysis was undertaken to identify barriers and facilitators for strengthening MLTC-M primary care according to the five health system pillars in the Lancet Global Health Commission on High-Quality Health Systems Framework.

**Results:**

The literature search yielded 4322 citations, evaluated 202 studies and identified 36 for inclusion. Key barriers within the people pillar included poverty, low health education and low health literacy; within the platform pillar, fragmented services and lack of multimorbid care guidelines were mentioned; within the workforce pillar, lack of required skills and insufficient health workers; and in the tools pillar: a shortage of essential medicines and adverse polypharmacy effects were prominent. A lack of political will and the absence of relevant national health policies were identified under the governance pillar. Facilitators within the people pillar included enhancing self-management support; within the platforms, pillar included integration of services; within the tools pillar, included embracing emerging technologies and information and communication technology services; and governance issues included upscaling interventions to respond to multimorbid care needs through enhanced political commitment and financial support.

**Conclusions:**

Potential solutions to strengthening the healthcare system to be more responsive to people with MLTC-M include empowering service users to self-manage, developing multimorbid care guidelines, incorporating community health workers into multimorbid care efforts and advocating for integrated person-centred care services across sectors. The need for policies and procedures in LMICs to meet the person-centred care needs of people with MLTC-M was highlighted.

**STRENGTHS AND LIMITATIONS OF THIS STUDY**

- The involvement of an interdisciplinary research team that comprised experts in the subject under review helped to enhance quality.
- The review adopted an evidence-based, high-quality health systems framework proposed by the Lancet Global Health Commission in the Sustainable Development Goals Era to strengthen its findings and presentation.
- As with any review exercise, our search strategy may have missed relevant articles that were not published or readily available online.

## Background

 Multiple long-term conditions-multimorbidity (MLTC-M), generally understood as the coexistence of two or more chronic conditions in a single person,^1^ presents a rapidly growing challenge in the contemporary health and social care systems across the globe.^2^ MLTC-M prevalence varies enormously depending on the age range being considered and the included conditions. This is supported by available evidence which shows that MLTC-M global prevalence estimates range from 37.2% (in the general population) to 95.1% (among 65 years and older).^3^ A recent literature review confirmed a comparable burden in low-income and middle-income countries (LMICs), ranging from 3.2% to 90.5%.^4^ MLTC-M consists predominantly of non-communicable diseases such as hypertension, cancer, cardiovascular diseases, respiratory conditions and mental health conditions.^3 5^ Two chronic infectious diseases that exacerbate the burden of MLTC-M, in LMICs, are HIV/AIDS and hepatitis C.^5 6^ MTLC-M service users have higher rates of mortality, polypharmacy, poor quality of life, higher healthcare costs, productivity loss and poor healthcare outcomes compared with service users with a single disease.^7 8^ Efforts to address the growing global burden of MLTC-M need a serious investment in health systems strengthening, which are constantly challenged by the evolving public health demands, especially in LMICs.^8^

The growing burden of MLTC-M has become a significant concern to health policy-makers and healthcare providers in LMICs where human and financial resources are limited, and the health system is failing to meet the ever-increasing healthcare needs of communities.^46^,^8^ Over the past decade, there has been a constantly expanding epidemiological transition from acute to chronic disease dominance in LMICs.^9^ Populations are ageing, with MLTC-M also occurring in younger age groups due to rapid nutritional transition.^10^ The vertical and programmatic organisation of health services in LMICs leave them ill equipped to address this growing burden. Coupled with the scarcity of resources to respond effectively, this puts additional pressure on health systems.^8 11^

An accepted response to improving MLTC-M outcomes includes adopting patient-centred or person-centred care approaches. While disease-centred care almost solely centres around disease management, person-centred care recognises the dynamics of the person’s experienced health problems.^12^ According to the Health Foundation, person-centred care models prioritise dignity and respect, integrated healthcare services, provision of targeted care and support, and empowering individuals to manage their own health.^13^ Similarly, the WHO 2007 defines person-centred care as holistic care responsive to a person’s biopsychosocial needs and values, thus understanding the person in their context.^14^ Interest in person-centred care for people with MLTC-M has grown due to the increasing realisation that health reform efforts ought to put people and communities at the centre of healthcare priorities.^12 14^ The literature emphasises person-centred integrated care for people with MLTC-M being more effective than verticalised single-disease care approaches.^15^ However, concerns about its low adoption among LMICs still exist.

Along with the person-centred care model, high-income countries (HICs) have employed the 3D approach to foster care continuity, coordination and a multifaceted holistic care approach for people with MLTC-M.^16^ However, there is broad consensus about the essential elements that reflect the utility of a person-centred care approach, such as encouraging self-management, shared decision-making with service users and deciding on a targeted care plan.^14 17^ These modifications necessitate a redesign of the health system in LMICs, which can be supported by the existing evidence regarding the opportunities and barriers to its successful implementation.^15^,^18^

Towards this end, the need to strengthen health systems for person-centred MLTC-M care in LIMCs is ever-increasing. To our knowledge, there is no systematic assessment of literature focusing on person-centred care interventions for MLTC-M in LMICs. This scoping review, therefore, aimed to identify barriers and facilitators for strengthening health systems for person-centred care and treatment of MLTC-M service users at the primary healthcare (PHC) level in LMICs. The review would identify and summarise pertinent evidence to assist policy-makers in advancing person-centredness in PHC multimorbid care in LMICs and beyond.

## Methods

Scoping reviews map the main concepts underpinning a research area to the available evidence. They are mainly used to identify gaps in the field and suggest possible recommendations to guide future research and interventions. This scoping review was guided by the methodological framework proposed by Arksey and O'Malley,^19^ which consists of five key steps:

1. Identifying the research question.
2. Identifying relevant studies.
3. Selection of eligible studies.
4. Charting the data
5. Collating and summarising the results.

### Patient and public involvement

This evidence synthesis relied on previously published articles. Service users and the broader public were not involved in the review process. However, the investigator team was constantly engaged during weekly meetings where presentations were made, and feedback opportunities provided.

### Identifying research questions

This scoping review was nested in an EvideNce led co-created HeAlth systems interventioNs for MLTC-M carE (ENHANCE) study that was being conducted in two provinces of South Africa to co-develop and evaluate an integrated and person-centred approach to the management of MLTC-M in South African PHC. The current review forms part of a formative work of the ENHANCE study which aimed at informing project-supported health systems strengthening efforts to optimise MLTC-M care. Further, the findings will provide recommendations for such efforts more broadly in LMICs.

The primary research question was, ‘What are the barriers and facilitators for strengthening health systems for person-centred care of people with MLTC-M at the PHC level in LMICs?’. This was formulated during the protocol development phase of the project, based on preliminary literature reviews and iterative consultations with the broader ENHANCE research team.

### Identifying relevant studies

A systematic search for available literature in the research area was conducted using the following electronic databases: EBSCOhost ((Academic search—ultimate, Academic search complete, MEDLINE, Health source (nursing academic edition), Health Source—(consumer edition)), PubMed and Google Scholar. The development of a comprehensive search approach was supported by subject matter experts who were members of the research team, employing specific medical subject headings (MeSH) words and phrases to identify pertinent articles on the subject. Keywords and MeSH terms were gathered and combined with Boolean operators such as, ''OR'' and ''AND'' as needed. DZ and GBB searched and reviewed papers that provided evidence on the barriers and facilitators for strengthening health systems for person-centred multimorbid care. The review focused on studies published from January 2010 to December 2023. The search strings consisted of the following combined keywords or MeSH terms: “person-centred OR people-centred OR patient-centred OR differentiated care OR complex care OR integrated care OR guided care OR collaborative care OR chronic care AND multiple chronic conditions OR multimorbidity OR comorbidity OR multiple long term conditions OR multiple illness OR non-communicable diseases Or complex conditions OR HIV OR AIDS OR Chronic Obstructive Pulmonary Disease OR cardiovascular disease OR asthma OR diabetes OR hypertension OR stroke OR depression OR anxiety OR chronic arthritis AND primary health care OR primary care OR public health care OR community care AND health system or health services or healthcare system AND low and middle-income countries or developing countries OR (the list of all World Bank LMICs)”.

The search strategy was piloted to check the appropriateness of keywords and databases. The comprehensive search strategy and the respective databases are provided in online supplemental table 1.

### Selection of eligible studies

All the papers obtained from the search process were imported into and screened for eligibility using Ryann, a web and mobile application for systematic reviews.^20^ The title and abstract screening were conducted by two authors (DZ and GBB), guided by the Population–Concept–Context framework recommended by the Joanna Briggs Institute (JBI),^21^ illustrated in table 1. The review focused on research concerning persons simultaneously suffering from more than one long-term condition. This included several expressions of the phrase MLTC-M based on titles and abstracts of preliminary literature reviews. Several concepts and paradigms related to person-centred care were further included in the search strings. The review also included other studies that addressed crucial person-centred interventions such as integration of healthcare services, self-management support and collaborative decision-making. Eligibility criteria ensured that the content of the included studies was relevant to the research question. DZ and GBB reviewed the full text and extracted data from the papers that met the inclusion criteria.

**Table 1:** Framework for determining the eligibility of studies for inclusion

Population	Studies focused on people diagnosed with MLTC-M
Concept	Barriers and facilitators for strengthening health systems for person-centred MLTC-M care.
Context	Primary healthcare, low-income and middle-income countries.

MLTC-Mmultiple long-term conditions-multimorbidity

### Eligibility criteria for the included studies

Articles were included or removed based on predefined eligibility criteria which were sufficiently outlined to incorporate all potentially relevant papers. In this review, the researchers limited the sources of evidence to peer-reviewed primary research studies and literature reviews to include reliable information and ensure evidence quality.

#### The type of population

The review included publications that explored all population categories with two or more chronic conditions concurrently occurring. Studies were included if they reported on PHC and community-based health systems within the LMIC context (as defined by the World Bank).^22^ The review was limited to LMIC articles to complement the Lancet Global Health framework^23^ built using data gathered from LMIC settings.

#### Type of interventions

The review covered all works on health systems that addressed aspects of person-centred care such as service users’ engagement and empowerment, service integration, self-management support, community-based interventions, shared decision-making and self-care.^15^

#### Type of outcomes

Outcomes were categorised and documented following the five essential pillars of the health system as defined by the Lancet Global Health framework.^23^ The framework emphasised high-quality health systems, including how health systems impact health outcomes such as quality of life, access to healthcare, the efficiency of services and the overall health system performance.

#### Time frame

Publications in English from January 2010 to December 2023 which focused on person-centred MLTC-M care were reviewed.

Studies published in other languages than English and conducted in HICs (as defined by the World Bank,^22^) and published before January 2010 and after December 2023 were excluded.

The reviewers met weekly to discuss their findings and agreed on articles to be included in the study. Disagreements were resolved by consensus. It was planned that a third author, AJvR, would resolve significant discrepancies if DZ and GBB could not reach a consensus. Duplicates were removed with Ryann software. Ultimately, 36 papers met the inclusion criteria and were included in the study. The screening results were reported as conceptualised by the Preferred Reporting Items for Systematic Reviews and Meta-Analyses extension for Scoping Reviews (PRISMA) checklist and mapped using the PRISMA-Protocols chart.^24^

### Charting the data

A data charting spreadsheet was developed using Microsoft Excel (V.2019) to capture all relevant information from the included studies. The data extraction form was piloted by two reviewers (DZ and GBB), using five articles to ensure consistency. A summary table of the extracted data was produced following the JBI manual for evidence synthesis format: first author name and year of publication, country/study setting, study aim/purpose, population, and sample, and the key findings (barriers and facilitators), as indicated in online supplemental table 2. To ensure consistency in data gathering and processing, two independent reviewers and a third author (when necessary) met individually to discuss challenges encountered in data collection. These results were presented according to the research question, the overall purpose of the review and the adopted conceptual framework. Gaps for strengthening health systems for person-centred multimorbid care were identified and reported.

### Collating, summarising and reporting the results

**Figure 1:**
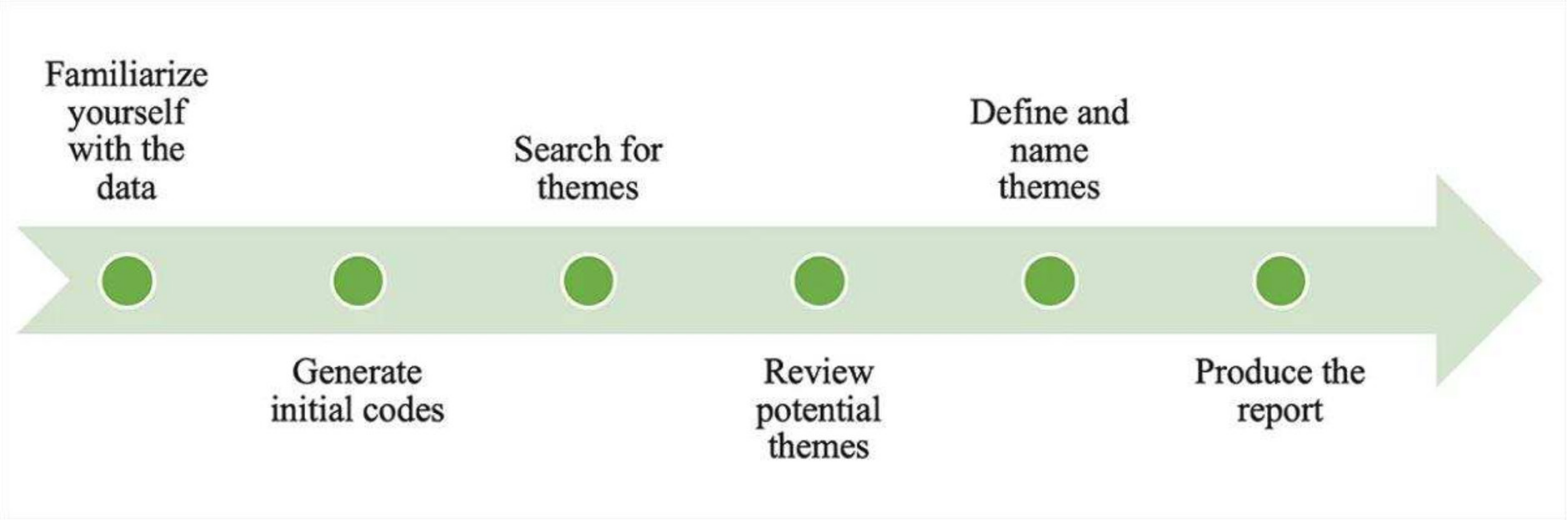
A six-step process of the thematic analysis of collected data: adapted from Braun and Clarke.^24 25^

The pillars of a framework from the Lancet Global Health Commission on High-Quality Health Systems in the Sustainable Development Goals Era^23^ guided a descriptive qualitative content analysis of the collected data. Data were accordingly deductively coded and organised into the following key pillars: people, workforce, governance, platforms and tools. Figure 1 shows the adopted six-step data analysis process proposed by Braun and Clarke.^25^ Common themes were charted and presented under the barriers and facilitators categories.

As illustrated above, the first step involved data familiarisation, during which the first author (DZ) read all 36 papers that met the inclusion criteria in detail. The second step involved the deductive and inductive generation of thematic codes. The initial codes were then transformed into emergent themes in the third step. Fourth, the themes were rigorously reviewed to summarise all emerging themes from the data set. The fifth step involved two authors (DZ and GBB), ensuring the analysis was robust and consistent. This was also verified by a third author (AJvR) to triangulate themes before the report was produced in the final step.

## Results

### Details of the search and screening flow

**Figure 2:**
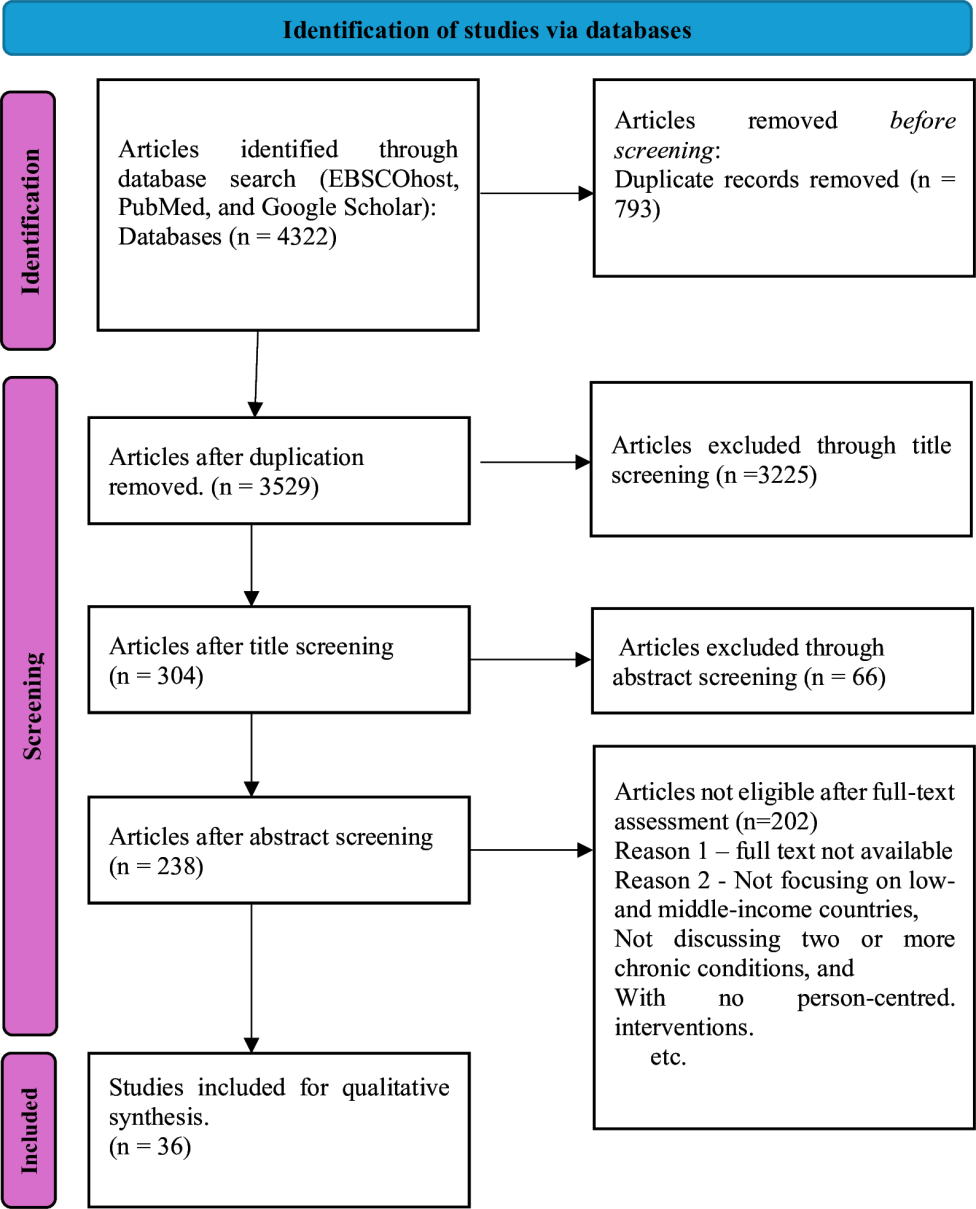
A PRISMA flow chart of the search results (Source: Braun and Clarke^24^). A summary of the process to identify eligible studies included in the scoping review. PRISMA, Preferred Reporting Items for Systematic Reviews and Meta-Analyses.

As shown in figure 2, our initial search strategy identified 4322 articles from online databases (EBSCOhost, PubMed and Google Scholar). After removing duplicates, we were left with 3529 articles. A total of 3225 articles were excluded after title screening, 304 abstracts were screened, and 66 articles were excluded for being undertaken in HICs, an absence of focus on person-centred care or not focusing on PHC. Of the 238 full-text articles we examined, only 36 met the inclusion criteria and were included in the analysis. Figure 2 shows the PRISMA flow chart of the search results.

**Figure 3:**
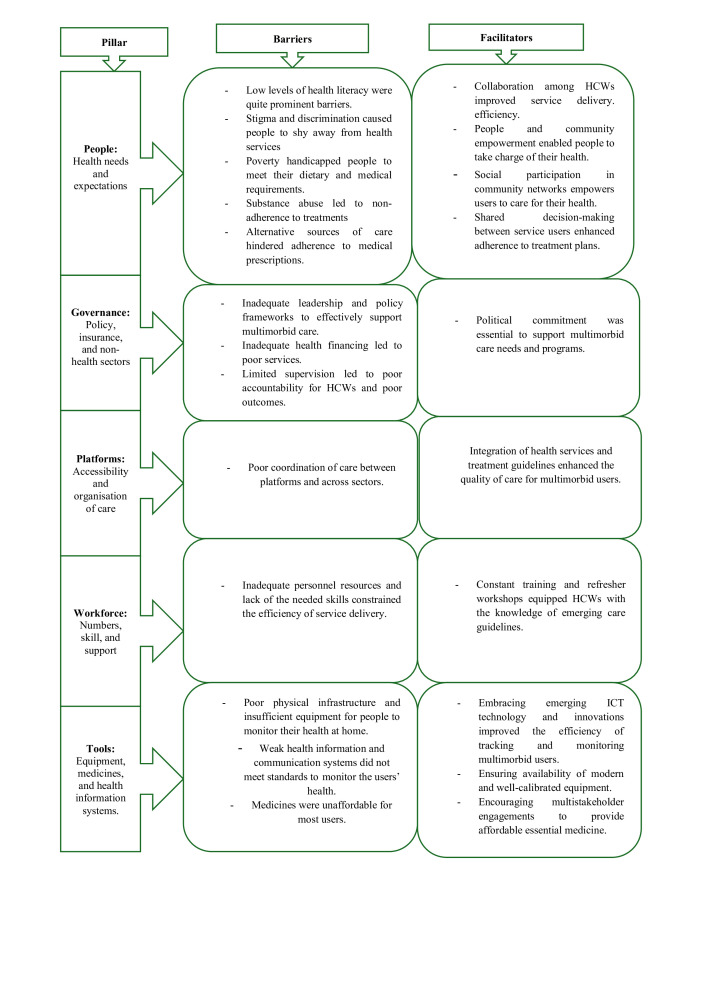
A summary of key themes identified per pillar during evidence synthesis for barriers and facilitators for person-centred multimorbid care in LMICs. HCWs, healthcare workers; ICT, information and communication technology; LMICs, low-income and middle-income countries.

This review examined the factors that supported and hindered primary health systems in LMICs from improving person-centred multimorbid care considering the people, governance, platforms, workforce and tools. A summary of key themes per pillar is illustrated in figure 3.

The review showed that less attention was paid to MLTC-M research in LMICs, with just a few countries, such as South Africa, India and Kenya, making significant efforts to increase it. Table 2 shows the distribution and the number of retrieved articles within the LMICs.

**Table 2:** Location of the studies included in the scoping review

Study location	Number of articles
South Africa	13
Mexico	1
Malaysia	1
Ethiopia	1
Brazil	2
Ghana	1
India	3
Lebanon	1
Malawi	1
Zimbabwe	2
Nigeria	1
Kenya	3
Botswana	1
Sub-Saharan Africa	2
More than 1 low-income and middle-income country	3
Total	36

The emergent themes and subthemes concerning the perceived barriers and facilitators for strengthening the health systems of people with MLTC-M are highlighted in online supplemental table 3.

### Pillar 1: people: health needs and expectations

A key aspect of person-centred care is consideration of the needs, preferences, experiences and other factors exhibited by individuals, families and communities.^23^ Several subthemes emerged concerning barriers and facilitators to care related to individuals and communities including traditional and cultural beliefs, stigma, poverty, low health knowledge and social participation.

#### Barriers

##### Poor health literacy

Poor health literacy and low education levels were reported as key barriers to effective service utilisation.^26 27^ Several studies concluded that low health literacy results in the late presentation of illnesses to facilities, which fueled the transmission of infectious diseases, deterioration of health and ultimately an increase in complications and mortality.^26^,^28^ Service users lacked the basic knowledge to actively engage in decision-making about treatment, self-management and health-enhancing behaviour modification.^2729^,^32^

##### Poor health-seeking behaviour

Four reviewed studies cited poor health-seeking behaviour and the impacts of providing effective care to people with MLTC-M.^2729^,^31^ This was attributed to various factors, including poor health literacy, unaffordable health services, and religious and cultural beliefs.^27 30^ Poor health-seeking behaviour was reported to compromise efforts to fight MLTC-M as some long-duration conditions are asymptomatic and only manifest when they have reached a severe stage, which is more costly to manage.^33^

##### Stigma and discrimination

Stigma and discrimination in the family or community were documented as barriers to health-seeking behaviour, disclosure or treatment adherence, leading to low uptake of healthcare services.^34 35^ Additionally, it was challenging to offer multimorbid care to patients on long-term treatment in communities due to stigma and discrimination, as some were not comfortable disclosing their positive status and obtaining the necessary care support.^29 36 37^ Reported stigma from healthcare services included the provision of substandard care, physical and verbal abuse, and longer waiting times for poor and other vulnerable minority subgroups for healthcare services.^37^

##### Poverty

Several studies report inadequate funds to buy food (to meet special diet needs) and essential medicines (where medicines were not freely provided), as well as transport costs to visit the PHC services, and this compromised adherence to appointments and health outcomes.^26 32 34 37 38^

##### Substance abuse

This review showed that substance abuse caused self-neglect in patients, which undermined multimorbid care efforts,^27 30^ exacerbating the progression of long-duration conditions due to addiction, self-neglect and non-adherence to medication.^30^

##### Alternative sources of care

Two studies reported that alternative sources of care, such as traditional and religious healing methods, compromised adherence, with some people completely replacing their biomedical medications with herbal medications.^30 39^ One study noted that conflicting information from biomedical and traditional service providers confounded efforts to empower service users to manage their health at the PHC level.^39^ This affected the patients’ lifestyle and approach to self-management as some patients preferred traditional services to biomedical treatment options because they costed less.^39^ Some religious and traditional teachings gave people a negative perception of biomedical treatments leading to non-adherence to treatments, hence frustrating health outcomes,^30 39^ although some evidence supported that the two were complementary.

### Facilitators

#### Collaborations

A treatment and care approach that included the active involvement of service users, families and a diverse team of specialised service providers in a coordinated manner was reported to enable care for people with MLTC-M, allowing for a wide range of thoughts and factors to be considered during treatment.^32^ Provider–service user collaborations (through a shared decision-making model) emerged as key for person-centred care as they promoted services that met the holistic needs of people, in addition to their immediate medical needs.^40 41^ Developing management and health promotion interventions collaboratively between healthcare providers and service users strengthened service users’ capabilities, opportunities and motivations resulting in sustained psychological support in the treatment journey, especially for service users with limited social support and those who face stigma.^29^ Several instances were noted where collaborations between health professionals and family members yielded positive care outcomes for people with MLTC-M.^41 42^

#### Individual and community empowerment

Enhanced health promotion programmes and education at individual and community levels were ideal opportunities to raise awareness about care for people with MLTC-M.^26^ Raising awareness through various advocacy strategies was an opportunity to encourage the utilisation and uptake of services.^42^ One study noted that adopting the WHO Innovative Care for Chronic Conditions (ICCC) Framework, in LMICs presented an opportunity to raise awareness of chronic conditions through public health advocacy, and co-creating environments that promoted long-term positive lifestyle decisions.^34 43^ Another study reported that harnessing the self-determination theory into person-centred care promoted feelings of being understood and cared for and encouraged people with MLTC-M to internalise the need to adopt healthy lifestyle behaviours.^44^ Community health workers (CHWs) were viewed as well-placed to empower service users with a sense of self-management, adequate knowledge of diseases and emerging services, and choice over health-related decisions due to the respect afforded to them, and their role within communities.^26 35 45^ Enough medication education for people with MLTC-M provided a chance to recognise and address the issues that were commonly brought up (such as taking medications incorrectly in terms of dose, frequency and timing of meals) to achieve optimal clinical outcomes and to reduce side effects.^43 45^ Health advocacy agencies were encouraged to leverage the usual and routinely conducted community gatherings to drive health education and promotion programs.^26^

#### Social participation

Five studies reported strengthened social participation in person-centred care to improve outcomes.^31 34 40 42^ Two studies reported that community-supported management (provision of service user and family support pathways within communities) of MLTC-M might be a more sustainable model of care to achieve better health systems and service outcomes.^40 43^ Another study highlighted the need for people with MLTC-M to put more effort into improving social interaction and engaging with others, especially through establishing community networks and adherence clubs to support each other.^42^ Encouraging the involvement of the service users’ social networks in promoting adequate health education and self-management enabled people with MTLC-M to take charge of and make better decisions about their conditions.^30 43^ Evidence shows the need for effective provision of practical guidance on self-management and lifestyle modification strategies, as well as structured, person-centred education, as key for people to control their health.^31 32 36 43 44^ There was consensus for a need to address social factors such as promoting positive stress management strategies and providing sufficient emotional and family support.^29 30 34 40 43^

#### Shared decision-making

Providing platforms for shared decision-making opportunities between service users and providers enabled people with MLTC-M to be more in control of their health.^31 37 43^ This approach included empowering service users with relevant information regarding their condition and treatment options, including possible risks, benefits, costs and significant uncertainties.^46^ Service users’ preferences, beliefs and values were respected and enhanced in the treatment and care management process, motivating them to adhere to advice and resulting in quality improvement.^30 43 47 48^

### Pillar 2: governance: policy, insurance, and non-health sectors

The WHO proposed that good health governance entails ensuring that strategic policy frameworks exist and are complimented with effective programmes, attention to system design and accountability.^49^ In this current review, several subthemes emerged, including policy frameworks, accountability, health financing, building research capacity and adopting contemporary leadership models for multimorbid care.

#### Barriers

##### Leadership and policy frameworks

The review suggested that effective management of MLTC-M was impeded by the limited political commitment to prioritise the management of MLTC-M in national health strategies, plans and policies.^30 48^ Literature has confirmed that most health organisational cultures were unsupportive or not congruent with person-centred care, resulting in poor health service delivery for people with MLTC-M.^32 50^ In most settings, policy and research were single-disease oriented, and the need to realign them to inform efforts and response strategies against the MLTC-M burden received less attention.^43^

##### Poor health financing

Financial resources are key to promoting quality improvement. However, a lack of financial resources was believed to impede the effective implementation of the suggested multimorbid care initiatives, such as supporting outreach for community health promotion programmes and procuring essential medicines and other equipment for monitoring patients’ health at home.^29 30 43 48^ Most nations cited the poor functioning of national health insurance programmes and the absence of clear health funding models as major barriers to accessing health services and improving the quality of multimorbid care.^35 41 43^ While some governments were making efforts to make essential medications cheaper for service users by covering them through social health insurance policies to make them more affordable to service users, in some countries, these models were not operating at their best due to economic hardships.^32^

##### Poor supportive supervision and accountability

Poor leadership and supervision were reported to result in low uptake and implementation of emerging guidelines, services and policies for caring for people with MLTC-M—ultimately compromising health service delivery.^32^ The commonly reported drivers of weak leadership and poor management included a shortage of skilled human resources, insufficient coordination of care, inappropriate fit of policies, low uptake of policies and programmes, and poor accountability.^34^ Lack of accountability was reported at various levels in the healthcare system, as most countries and institutions had no clearly defined accountability models in place, which greatly compromised quality improvement.^35 44^

### Facilitators

#### Political commitment

Stakeholders’ buy-in emerged as an important driver for effective policy formulation and implementation of emerging practices and policies for person-centred multimorbid care.^29^ Good leadership skills at low managerial levels were also reported to facilitate quality and effective counselling of people with MLTC-M, which focused on measuring the outputs and improving the quality of care.^32^ Literature reported a growing political commitment to developing and supporting more integrated and holistic person-centred care reforms that effectively addressed the needs of people with MLTC-M.^29 50^ In some settings, person-centred care for people with MLTC-M was pivotal in the PHC transformation process and this gained much-needed support from governments and key stakeholders.^29 32^

#### Learning and improvement

Evidence showed the need for continuous research and training—especially implementation science research,^51^ to increase knowledge and inform policy-makers and stakeholders to develop data-driven and innovative policy interventions to improve service delivery. The need for continuous review of existing policies and guidelines to include emerging person-centred care services and technologies for managing MLTC-M users was identified.^30 43 44^ The need for consistent supportive learning and encouraging community participation in the policy formulation and evaluation processes to promote ownership of the policies and programmes was reported by several studies.^30 31 43 52^ A literature review noted that most countries took time to learn, review and align healthcare policies with emerging best practices, which hindered efforts to efficiently provide responsive health systems as healthcare needs kept evolving with time.^53^ The need for sensitivity to people’s values and culture was highlighted.^29 42^ Improving clinical governance through shifting from hierarchical to bottom-up approaches and more shared decision-making had positive leadership implications and promoted quality improvement.^32 43^ This was achieved through continuous supportive learning of emerging guidelines.^44^

### Pillar 3: platforms: accessibility and organisation of care

One key indicator of a well-performing health system is good coordination of services around the health needs and demands of people.^23^ This entails good care organisation, the integration of health providers within and across healthcare settings, the development of clear referral guidelines and well-performing outreach programmes. Guided by this pillar, several subthemes emerged, relating to barriers and facilitators to strengthening the health system towards improving person-centred care for people with MLTC-M.

#### Barriers

##### Poor organisation of care

There was a reported paucity of person-centred and integrated care perspectives and approaches to addressing MLTC-M in most LMICs, especially between physical chronic conditions and mental disorders.^26 29 35 40^ Further, poor coordination of care between different service providers (with no full understanding of the conditions’ history and progression) caused anxiety and uncertainty for service users.^43 47 52^ Additionally, fragmented care models that emphasised verticalised and disease-focused treatment methods were very prevalent in some areas. These models were less responsive to the growing demands of multimorbid service users and did not support continuity of care.^29 30 35 42^ Lack of standardised referral, appointment or treatment guidelines across settings contributed to poor health management and care.^53 54^ Inaccessibility to PHC facilities was also a notable barrier to the quality of care.^43 48^ Furthermore, it was discovered that many people sought assistance from highly priced private medical institutions and pharmacies due to the low quality of care provided in the public sector in some nations.^43 55^

### Facilitators

#### Integration of health services

Decentralised and adequately equipped facilities (in terms of infrastructure readiness and provider preparedness) were important for improving access to early initiation of treatment and high-quality continued multimorbid care.^27 35 40 54^ Operational factors that promoted healthcare utilisation and outcomes, including information sharing, service integration, organisation and interpersonal interactions, were quite helpful.^55 56^

The need to integrate vertically organised treatment approaches to improve the quality-of-service delivery emerged as important,^26 31 36 52^ as did coordination of care within and across sectors.^39 40 51^ Improved information flows to bridge information gaps^42^ assisted with opportunities for leveraging existing health information systems (HIS) infrastructure from other programmes such as the HIV programmes.^31^ Family support pathways^41^ and health promotion interventions, such as raising awareness of MLTC-M through social media platforms or ‘adherence clubs’, were identified.^33^

Several factors should be addressed to improve the service quality for people with MLTC-M in PHC settings. These factors included improving community-supported self-management systems, improving utilisation of available services, intensifying health education through community outreach, embracing an integrated approach to chronic disease management, developing referral system guidelines, integrating treatment and palliative care services.^2630 31 36 39 42 50 51 55^,^59^ The WHO ICCC model was identified as ideal for meeting the growing needs of people with MLTC-M.^54^

### Pillar 4: workforce: numbers, skill, and support

Special attention needs to be taken to ensure that the health workforce is adequately and equitably distributed, equipped with an appropriate skills mix and has supportive working environments to perform at their best and sustainably satisfy the health demands of the expanding population.^60 61^ In this review, several subthemes emerged, which covered barriers and opportunities to strengthening the health workforce and reorienting the model of care towards improving person-centred care for people with MLTC-M, including healthcare workers (HCWs) training and motivation, equitable distribution of HCWs and creating a conducive working environment.

#### Barriers

##### HCW resources

In most settings, the following barriers were noted: providers with inadequate clinical skills for MLTC care, lack of training for person-centred care, insufficient human resources, language barriers for foreign health providers and lack of motivation due to poor remuneration.^26 34 43 48 62^ Previous studies reported that service provision in LMICs was confronted by human resource deficiencies, with healthcare personnel who could offer quality services leaving for better opportunities.^63^,^70^ Poor staff-service user ratios, poor working conditions and poorly capacitated staff resulted in work-related stress due to heavy workloads.^54 55 58^ Language barriers between providers and users also emerged as a challenge in some contexts.^30^

### Facilitators

#### Training and capacity building programmes

Several studies described the value of continuous training for CHWs to support service users in decision-making and self-care as a facilitator of improved health outcomes.^30^,^3241 43 71 72^ Training the caregivers could also enhance capacity and efficiency in providing the needed care skills.^3273^,^76^ In some settings, the health systems established other avenues to support persons living with MLTC-M through club-based models where retired healthcare professionals supported the continuous treatment of MLTC-M.^33 77 78^ In sub-Saharan Africa, previous and existing HIV prevention efforts created a broad range of key community structures and a knowledgeable workforce capable of communicating effective HIV messages that could be harnessed to scale up integrated MLTC-M care efforts.^44 79^ Training in person-centred care approaches should be incorporated into ‘nurses’, ‘doctors’ and other PHC providers’ basic curricula to provide a stronger learning foundation.^31 32 80^ The existing CHWs should be well equipped with person-centred care principles so they can effectively leverage the positive reception they are given in the communities to conduct numerous awareness programmes that enhance good health-seeking behaviours.^39^ The study also showed that CHWs took advantage of their proximity to the communities they serve to reduce social barriers and increase service adoption because they were better suited to bridge the gap between communities and PHC facilities.

### Distribution of service providers

PHC service providers were a vital element in health service provision. However, according to previous reports, even in nations where the number of PHC service providers was unaffected, their distribution was inequitable, especially in rural settings.^30^ Studies recommended the equitable distribution of existing PHC service providers across settings and continuous training of emerging person-centred care skills for multiple services compared with narrowly defined task training.^4 29 34 81^ Additionally, task-sharing between HCWs and CHWs alleviated the human resource shortage experienced in PHC facilities.^26 35 51^ Therefore, CHWs should be trained to scale up their responsibilities and effectively deliver person-centred care services to people with MLTC-M through a multitasking approach.^4 42 55^

### Pillar 5: tools: equipment, medicines and HIS

Health tools are equipment and resources that enhance the efficient operation of health systems, such as healthcare facilities, supplies, medicines and HIS.^49^ Several subthemes surfaced in the current review, including prospects and obstacles to enhancing person-centred care for individuals with MLTC-M, such as resource constraints and physical infrastructure, as well as emerging technology and innovations.

#### Barriers

##### Poor physical infrastructure and equipment

Several reviewed studies indicated that the infrastructure and equipment in most PHC facilities were not conducive to care for MLTC-M conditions.^30 52^ In particular, there was mention of inadequate, outdated or lack of essential equipment such as blood pressure monitors, and an erratic supply of resources like electricity and water.^26 30 36 82^ Inadequate private areas for the needed counselling services within facilities hampered service delivery efforts.^83^

##### Health information and communication systems

Four studies reported that most LMICs responded to the needs of multimorbid users inefficiently due to poor health information and communication systems.^26 29 30 43^ A scoping review of 30 articles focused on LMICs reported that the health information and communication (HIS) systems in most of these settings were fragmented and not reliable.^31^ Outdated and poorly managed paper-based recording systems hampered efforts for effective data-driven policies and practices.^42^ Poor communication systems were widely reported as a major impediment to the quality of service delivery at the PHC level.^26 29 37 43^ Poor communication occurs at different levels varying from a lack of person-centred communication,^26^ poor communication of results to service users and the need for tests,^43^ and poor communication about the prescribed drugs,^37^ compounded challenges for the continuity of care and management of people with MLTC-M.

##### Medicines

Drug burden was reported as a key barrier to multimorbid care. This took many forms: medication side effects (such as eczema, rashes, weight gain, frequent urination and hair loss), shortage of medicine, shortage of financial resources to procure essential medicine, adverse reactions due to multiple fragmented prescriptions, and lack of care coordination.^27 31 36 50 55 59 82 83^ Polypharmacy was reported to result in treatment fatigue, leading to poor treatment adherence and poor health outcomes.^33 50^

### Facilitators

#### Information and communication technology and innovations

Mobile Health (mHealth) applications provided immense opportunities to improve the care of people with MLTC in LMICs. These ranged from mobile telemedicine for screening and monitoring, mobile phones to enable phone-based home glucose monitoring for people with diabetes, reminders to service users for their clinic dates, capturing chronic disease data electronically to continuously monitor patient progress and drug collection, implementing shared electronic medical records between providers at different levels of care and mobile learning (m-Learning) to provide online training to service providers in remote settings.^26 30 42 59 60^ Information and communication technology (ICT) was widely reported to promote person-centred care, increase quality healthcare, and facilitate information and health education sharing, between providers and service users through enhanced online interaction, which also minimised travel time and cost.^30 82^

#### Equipment and essential medicines

Highlighted was the need to develop policies and efficient systems for procurement, use and management, including the calibration of equipment and technologies.^34 35 41 43 45^ Multistakeholder engagement and partnership were highly recommended to provide essential medicines at affordable and readily accessible rates.^45^

## Discussion

To the best of our knowledge, this was the first scoping review to look at health system factors that influenced person-centred care for MLTC-M in LMICs, and in particular, on how to strengthen the pillars of LMICs health systems to enable the care of people with MLTC-M at the PHC level. Barriers were more commonly described than facilitators and occurred across all five pillars. Poor health-seeking behaviour, stigma and discrimination, low health literacy, poor socioeconomic status, poor adherence to drugs, fragmented drug prescriptions, and use of alternative sources of care were major barriers to engaging and empowering people and communities to become part of the PHC person-centred care plans for MLTC-M in LMICs.^2729^,^34 36 39^ Some of these barriers, for instance, fragmented drug prescriptions and poor adherence to drugs were also reported by studies in high-resource settings,^62 63 70 72^ It was reported that developing practical guidelines on self-management support and lifestyle modification strategies, as well as structured person-centred education, empowered people and the communities to be in control of their health.^35 43 44 64 65^ Promoting sustained relationships between service providers and service users (through a shared decision-making approach) to ensure continuity of care was deemed helpful.^40 66 69^ This was supported by a previous study that noted the impossibility of achieving optimum healthcare benefits when service user self-care goals were not included in multimorbid treatment plans.^79^

Sound governance is an essential factor in providing a more responsive health system for people with MLTC-M. However, evidence confirmed that health delivery was largely compromised by several governance setbacks, namely lack of political commitment to prioritise multimorbid care, poor health financing models, poor adoption and implementation of emerging policies and guidelines, and inadequate financial resources.^29 36 40 42 43^ Previous evidence supports the current findings that government and stakeholder buy-in and involvement in person-centred policy formulation and implementation increase access to healthcare.^69^ Good leadership and accountability issues were the key facilitators of an effectively performing PHC system. The review identified the need for continuous assessment and transformation of the PHC to include emerging person-centred services and technologies to respond to the complex needs of people with MLTC-M.^29^ Adopting and effectively implementing pro-PHC strengthening policies, guidelines, and strategic plans, decentralising leadership services, and equitable distribution of resources through appropriate health financing modalities demonstrated a quality improvement effect.^70^,^72^ Complementary to the existing evidence, a previous study reaffirmed that multisectoral partnerships were ideal for enhancing health system capacity.^75^

Under the platform pillar, the review noted the importance of good coordination of services around people’s health needs and demands as a vital indicator of a well-performing health system. However, this was hampered by poor coordination of care and verticalised care models that emphasised disease-focused treatment approaches.^48 70 84^ This assertion was supported by a study in HICs that reported that despite the usefulness of clinical guidelines for single conditions, there was an abundance of them in general practice and following them all could lead to considerable polypharmacy.^69^ Further, the lack of standardised referral, appointment and treatment approaches for people with MLTC-M was also highlighted.^54^ Several facilitators that enhance health systems’ performance towards care for people with MLTC-M were also reported. The facilitators included decentralised and adequately equipped facilities as well as integration of services.^27 35 40 84^ This was strongly supported by an earlier review that found that seeing numerous health providers through a verticalised model of care exacerbates the difficulty of managing medicines and harmonising advice among providers. As the MLTC-M burden grew, treatment coordination became increasingly complex and necessary.^79^,^81^

The health workforce is a key component of a well-performing health system. However, health workforce functioning for person-centred multimorbid care at PHC settings in LMICs is faced with various challenges, such as limited clinical training and knowledge updates for providers, language barriers for foreign health providers due to their inability to speak in local languages (which service users could understand) and poor staff-service user ratios.^26 42 43 52 54 58^ While there were several barriers to strengthening person-centred care for people with MLTC-M, considerable opportunities for its improvement were also reported. Task-sharing with lower-level service providers such as CHWs^38^ and in-service training of PHC service providers to remain up to date with emerging integrated chronic care approaches were emphasised.^26 29 31 73 78 79^ Furthermore, other studies recommended the alignment of the professional training curricula of PHC healthcare providers such as doctors and nurses to equip them to provide person-centred multimorbid care.^3670 82^,^84^

Concerning tools, limited access to evidence-based guidelines and protocols for MLTC-M management, poor supply chains and capacity to supply essential medicines and a lack of coordinated integrated HIS for people with multimorbidity emerged as challenges.^27 31 33 43 45^ Our findings supported instituting coordinated HIS mechanisms to keep close track of people with MLTC-M, embracing emerging ICT services to support record keeping, results dissemination and communication with service users, decentralisation of health facilities and essential medicines, and developing supply chains and national procurement policies as vital solutions to enhance health delivery for multimorbid care systems.^33 36 41 43^ Similarly, earlier studies suggested that emerging ICT systems and technologies should be integrated into existing primary multimorbid care systems to strengthen communication and coordination of care across providers.^75^,^78^

### Strengths and limitations

The main limitation of this scoping review was the lack of uniformity in defining disease combinations that characterise a multimorbid state using the term ‘MLTC-M’. This limited the potential to gather all the studies that could be relevant to this study. However, we found that in most cases where the term ‘MLTC-M’ was used to search, the term ‘comorbidity’ also appeared in the list of keywords. We deliberately added the terms ‘chronic disease and comorbidity’ to circumvent the problem. Because the review was limited to articles published in English, it is possible that relevant articles published in other languages were overlooked.

## Conclusion

This evidence synthesis provided important insight into an array of barriers and facilitators to person-centred multimorbid care at the PHC level in LMICs. Potential solutions to strengthening the primary health system to be more responsive to people with multiple chronic conditions included empowering service users to self-manage, developing multimorbid care guidelines, incorporating CHWs into multimorbid care efforts, ensuring adequate calibrated equipment and advocating for integrated person-centred care services across sectors. A commitment to invest in the adaptation and implementation of evidence-based guidelines is recommended because there was a notable pattern of poor adoption of suggestions. Context-specific research across settings is important and necessary for tailored policies and implementation strategies to strengthen health systems for person-centred multimorbid care.

## supplementary material

10.1136/bmjopen-2024-087451 online supplemental file 1

## Data Availability

All data relevant to the study are included in the article or uploaded as online supplemental information.

## References

[1] Head A, Fleming K, Kypridemos C (2021). Multimorbidity: the case for prevention. J Epidemiol Community Health.

[2] van Blarikom E, Fudge N, Swinglehurst D (2023). The emergence of multimorbidity as a matter of concern: a critical review. Biosocieties.

[3] Nguyen H, Manolova G, Daskalopoulou C (2019). Prevalence of multimorbidity in community settings: A systematic review and meta-analysis of observational studies. J Comorb.

[4] Abebe F, Schneider M, Asrat B (2020). Multimorbidity of chronic non-communicable diseases in low- and middle-income countries: A scoping review. J Comorb.

[5] World Health Organisation (2021). “Noncommunicable diseases,” world health organisation report. https://www.afro.who.int/health-topics/noncommunicable-diseases#:~:text=Noncommunicable%20diseases%20(NCDs)%20kill%2041,%2D%20and%20middle%2Dincome%20countries.

[6] Owen N, Dew L, Logan S (2022). Research policy for people with multiple long-term conditions and their carers. J Multimorb Comorb.

[7] Soley-Bori M, Ashworth M, Bisquera A (2021). Impact of multimorbidity on healthcare costs and utilisation: a systematic review of the UK literature. Br J Gen Pract.

[8] McPhail SM (2016). Multimorbidity in chronic disease: impact on health care resources and costs. Risk Manag Healthc Policy.

[9] Schneider J, Algharably EAE, Budnick A (2021). High Prevalence of Multimorbidity and Polypharmacy in Elderly Patients With Chronic Pain Receiving Home Care are Associated With Multiple Medication-Related Problems. Front Pharmacol.

[10] Divo MJ, Martinez CH, Mannino DM (2014). Ageing and the epidemiology of multimorbidity. Eur Respir J.

[11] Mahomed OH, Asmall S, Freeman M (2014). An integrated chronic disease management model: a diagonal approach to health system strengthening in South Africa. J Health Care Poor Underserved.

[12] Starfield B (2011). Is patient-centered care the same as person-focused care?. Perm J.

[13] The Health Foundation (2016). Person-centred care made simple. https://www.health.org.uk/sites/default/files/PersonCentredCareMadeSimple.pdf.

[14] World Health Organisation (2007). People-Centred Health Care: A Policy Framework.

[15] Poitras M-E, Maltais M-E, Bestard-Denommé L (2018). What are the effective elements in patient-centered and multimorbidity care? A scoping review. BMC Health Serv Res.

[16] Mounier-Jack S, Mayhew SH, Mays N (2017). Integrated care: learning between high-income, and low- and middle-income country health systems. Health Policy Plan.

[17] Dowrick C (2018). Patient-centred care for multimorbidity: an end in itself?. Lancet.

[18] Khatri RB, Wolka E, Nigatu F (2023). People-centred primary health care: a scoping review. BMC Prim Care.

[19] Arksey H, O’Malley L (2005). Scoping studies: towards a methodological framework. Int J Soc Res Methodol.

[20] Ouzzani M, Hammady H, Fedorowicz Z (2016). Rayyan-a web and mobile app for systematic reviews. Syst Rev.

[21] Joanna Briggs Institute The Joanna Briggs Institute Reviewers’ Manual 2015 Methodology for JBI Scoping Reviews.

[22] World Bank (2019). World Bank Country and Lending Groups.

[23] Kruk ME, Gage AD, Arsenault C (2018). High-quality health systems in the Sustainable Development Goals era: time for a revolution. Lancet Glob Health.

[24] Page MJ, McKenzie JE, Bossuyt PM (2021). The PRISMA 2020 statement: an updated guideline for reporting systematic reviews. Syst Rev.

[25] Braun V, Clarke V (2006). Using thematic analysis in psychology. Qual Res Psychol.

[26] Matenge TG, Mash B (2018). Barriers to accessing cervical cancer screening among HIV positive women in Kgatleng district, Botswana: A qualitative study. PLoS ONE.

[27] Koros H, Nolte E, Kamano J (2023). Understanding the treatment burden of people with chronic conditions in Kenya: A cross-sectional analysis using the Patient Experience with Treatment and Self-Management (PETS) questionnaire. PLOS Glob Public Health.

[28] Balcha TT, Skogmar S, Sturegård E (2015). Outcome of tuberculosis treatment in HIV-positive adults diagnosed through active versus passive case-finding. Glob Health Action.

[29] Truppa C, Ansbro É, Willis R (2023). Developing an integrated model of care for vulnerable populations living with non-communicable diseases in Lebanon: an online theory of change workshop. Confl Health.

[30] Gupta-Wright A, Manabe YC (2019). Implementation science: point-of-care diagnostics in HIV and tuberculosis. Clin Med (Lond).

[31] Janse Van Rensburg A, Dube A, Curran R (2020). Comorbidities between tuberculosis and common mental disorders: a scoping review of epidemiological patterns and person-centred care interventions from low-to-middle income and BRICS countries. Infect Dis Poverty.

[32] Amu H, Darteh EKM, Tarkang EE (2021). Management of chronic non-communicable diseases in Ghana: a qualitative study using the chronic care model. BMC Public Health.

[33] Kamvura TT, Turner J, Chiriseri E (2021). Using a theory of change to develop an integrated intervention for depression, diabetes and hypertension in Zimbabwe: lessons from the Friendship Bench project. BMC Health Serv Res.

[34] Malan Z, Mash R, Everett-Murphy K (2015). Qualitative evaluation of primary care providers experiences of a training programme to offer brief behaviour change counselling on risk factors for non-communicable diseases in South Africa. BMC Fam Pract.

[35] Colizzi M, Lasalvia A, Ruggeri M (2020). Prevention and early intervention in youth mental health: is it time for a multidisciplinary and trans-diagnostic model for care?. Int J Ment Health Syst.

[36] Godongwana M, De Wet-Billings N, Milovanovic M (2021). The comorbidity of HIV, hypertension and diabetes: a qualitative study exploring the challenges faced by healthcare providers and patients in selected urban and rural health facilities where the ICDM model is implemented in South Africa. BMC Health Serv Res.

[37] Hugo JFM, Maimela TCR, Janse van Rensburg MNS (2020). The three-stage assessment to support hospital-home care coordination in Tshwane, South Africa. Afr J Prim Health Care Fam Med.

[38] Mohr E, Daniels J, Beko B (2017). DOT or SAT for Rifampicin-resistant tuberculosis? A non-randomized comparison in a high HIV-prevalence setting. PLoS ONE.

[39] Naidoo K, Van Wyk J (2019). What the elderly experience and expect from primary care services in KwaZulu-Natal, South Africa. Afr J Prim Health Care Fam Med.

[40] Abrahams N, Gilson L, Levitt NS (2019). Factors that influence patient empowerment in inpatient chronic care: early thoughts on a diabetes care intervention in South Africa. BMC Endocr Disord.

[41] Page-Shipp L, Voss De Lima Y, Clouse K (2012). TB/HIV integration at primary care level: A quantitative assessment at 3 clinics in Johannesburg, South Africa. S Afr J HIV Med.

[42] Rachlis B, Naanyu V, Wachira J (2016). Community Perceptions of Community Health Workers (CHWs) and Their Roles in Management for HIV, Tuberculosis and Hypertension in Western Kenya. PLoS ONE.

[43] Rzewuska M, Carolina Guidorizzi Zanetti A, Skea ZC (2021). Mental-physical multimorbidity treatment adherence challenges in Brazilian primary care: A qualitative study with patients and their healthcare providers. PLoS ONE.

[44] Brooke-Sumner C, Petersen-Williams P, Sorsdahl K (2022). Strategies for supporting the implementation of a task-shared psychological intervention in South Africa’s chronic disease services: qualitative insights from health managers’ experiences of project MIND. Glob Health Action.

[45] Patrick H, Williams GC (2012). Self-determination theory: its application to health behavior and complementarity with motivational interviewing. Int J Behav Nutr Phys Act.

[46] Murphy K, Chuma T, Mathews C (2015). A qualitative study of the experiences of care and motivation for effective self-management among diabetic and hypertensive patients attending public sector primary health care services in South Africa. BMC Health Serv Res.

[47] Thornicroft G, Ahuja S, Barber S (2019). Integrated care for people with long-term mental and physical health conditions in low-income and middle-income countries. Lancet Psychiatry.

[48] World Health Organisation (2016). "WHO Framework on Integrated, People-Centred Health Services.

[49] World Health Organisation (2016). Health system governance. https://www.who.int/health-topics/health-systems-governance#tab=tab_1.

[50] Juma K, Reid M, Roy M (2018). From HIV prevention to non-communicable disease health promotion efforts in sub-Saharan Africa: A Narrative Review. AIDS.

[51] Chua SS, Kok LC, Yusof FAM (2012). Pharmaceutical care issues identified by pharmacists in patients with diabetes, hypertension or hyperlipidaemia in primary care settings. BMC Health Serv Res.

[52] Peer N, de Villiers A, Jonathan D (2020). Care and management of a double burden of chronic diseases: Experiences of patients and perceptions of their healthcare providers. PLoS ONE.

[53] Uwimana J, Zarowsky C, Hausler H (2012). Engagement of non-government organisations and community care workers in collaborative TB/HIV activities including prevention of mother to child transmission in South Africa: opportunities and challenges. BMC Health Serv Res.

[54] Waghela K, Shah NN, Saha S (2018). Morbidity Pattern and Role of Community Health Workers in Urban Slums of Durg and Bhilai City of Chhattisgarh. Indian J Community Med.

[55] Nkhoma K, Ahmed A, Ali Z (2018). Does being on TB treatment predict a higher burden of problems and concerns among HIV outpatients in Kenya? a cross-sectional self-report study. AIDS Care.

[56] Wang Y-P, Nunes BP, Coêlho BM (2019). Multilevel Analysis of the Patterns of Physical-Mental Multimorbidity in General Population of São Paulo Metropolitan Area, Brazil. Sci Rep.

[57] Page-Shipp L, Voss De Lima Y, Clouse K (2012). TB/HIV integration at primary care level: A quantitative assessment at 3 clinics in Johannesburg, South Africa. S Afr J HIV Med.

[58] Pati MK, Swaroop N, Kar A (2020). A narrative review of gaps in the provision of integrated care for noncommunicable diseases in India. Pub Health Rev.

[59] Kamvura TT, Dambi JM, Chiriseri E (2022). Barriers to the provision of non-communicable disease care in Zimbabwe: a qualitative study of primary health care nurses. BMC Nurs.

[60] Koch G, Wakefield BJ, Wakefield DS (2015). Barriers and facilitators to managing multiple chronic conditions: a systematic literature review. West J Nurs Res.

[61] Duda-Sikuła M, Kurpas D (2023). Barriers and Facilitators in the Implementation of Prevention Strategies for Chronic Disease Patients-Best Practice GuideLines and Policies’ Systematic Review. J Pers Med.

[62] Clouse K, Motlhatlhedi M, Bonnet K (2018). “I just wish that everything is in one place”: facilitators and barriers to continuity of care among HIV-positive, postpartum women with a non-communicable disease in South Africa. AIDS Care.

[63] Chang AY, Gómez-Olivé FX, Payne C (2019). Chronic multimorbidity among older adults in rural South Africa. BMJ Glob Health.

[64] Birke H, Jacobsen R, Jønsson AB (2020). A complex intervention for multimorbidity in primary care: A feasibility study. J Comorb.

[65] Yamey G (2012). What are the barriers to scaling up health interventions in low and middle income countries? A qualitative study of academic leaders in implementation science. Glob Health.

[66] Jindal D, Gupta P, Jha D (2018). Development of mWellcare: an mHealth intervention for integrated management of hypertension and diabetes in low-resource settings. Glob Health Action.

[67] Tapia-Conyer R, Saucedo-Martinez R, Mujica-Rosales R (2016). Enablers and inhibitors of the implementation of the Casalud Model, a Mexican innovative healthcare model for non-communicable disease prevention and control. Health Res Policy Syst.

[68] Bitton A, Fifield J, Ratcliffe H (2019). Primary healthcare system performance in low-income and middle-income countries: a scoping review of the evidence from 2010 to 2017. BMJ Glob Health.

[69] Ngangue PA, Forgues C, Nguyen T (2020). Patients, caregivers and health-care professionals’ experience with an interdisciplinary intervention for people with multimorbidity in primary care: A qualitative study. Health Expect.

[70] Mechili EA, Saliaj A, Xhindoli J (2022). Primary healthcare personnel challenges and barriers on the management of patients with multimorbidity in Albania. Health Soc Care Community.

[71] Søndergaard E, Willadsen TG, Guassora AD (2015). Problems and challenges in relation to the treatment of patients with multimorbidity: General practitioners’ views and attitudes. Scand J Prim Health Care.

[72] Espinosa-González AB, Delaney BC, Marti J (2019). The impact of governance in primary health care delivery: a systems thinking approach with a European panel. Health Res Policy Syst.

[73] Almirall J, Fortin M (2013). The coexistence of terms to describe the presence of multiple concurrent diseases. J Comorb.

[74] van Niekerk L, Bautista-Gomez MM, Msiska BK (2023). Social innovation in health: strengthening Community Systems for Universal Health Coverage in rural areas. BMC Public Health.

[75] Warfield ME, Lorenz L, Ali HN (2022). Strengthening Community Participation by People With Disabilities in Community-Based Group Homes Through Innovative Action Research. Front Public Health.

[76] Bokhour BG, Fix GM, Mueller NM (2018). How can healthcare organizations implement patient-centered care? Examining a large-scale cultural transformation. BMC Health Serv Res.

[77] Abimbola S, Baatiema L, Bigdeli M (2019). The impacts of decentralization on health system equity, efficiency and resilience: a realist synthesis of the evidence. Health Policy Plan.

[78] Ranabhat CL, Kim C-B, Singh A (2019). Challenges and opportunities towards the road of universal health coverage (UHC) in Nepal: a systematic review. Arch Public Health.

[79] Schiøtz ML, Høst D, Frølich A (2016). Involving patients with multimorbidity in service planning: perspectives on continuity and care coordination. J Comorb.

[80] Yardley S, Cottrell E, Rees E (2015). Modelling successful primary care for multimorbidity: a realist synthesis of successes and failures in concurrent learning and healthcare delivery. BMC Fam Pract.

[81] Melchiorre MG, Lamura G, Barbabella F (2018). eHealth for people with multimorbidity: Results from the ICARE4EU project and insights from the “10 e’s” by Gunther Eysenbach. PLoS ONE.

[82] Mishu MP, Uphoff E, Aslam F (2021). Interventions for preventing type 2 diabetes in adults with mental disorders in low- and middle-income countries. Cochrane Database Syst Rev.

[83] Rabkin M, Melaku Z, Bruce K (2012). Strengthening Health Systems for Chronic Care: Leveraging HIV Programs to Support Diabetes Services in Ethiopia and Swaziland. J Trop Med.

[84] Sansbury GM, Pence BW, Zimba C (2023). Improving integrated depression and non-communicable disease care in Malawi through engaged leadership and supportive implementation climate. BMC Health Serv Res.

