# Supplementary material for: Barriers and facilitators for strengthening primary health systems for person-centred multimorbid care in low-income and middle-income countries: a scoping review
Source: BMJ Open. 2024 Nov 27;14(11):e087451. doi: 10.1136/bmjopen-2024-087451 (PMC11603689; doi:10.1136/bmjopen-2024-087451)
Supplement: online supplemental file 1 [file bmjopen-14-11-s001.pdf]

Barriers and facilitators for strengthening primary health systems for person-centred multimorbid care in Low- and Middle-Income Countries: A scoping review.

Research Question:

"What are the barriers and facilitators for strengthening health systems for person-centred care of people with MLTC-M at the PHC level in LMICs?"

Identifying relevant studies

Online Supplemental Table 1*: A comprehensive search strategy and the respective databases and outcomes*

Database	Search terms applied	Search date	Filters	Number of articles
EBSCOHOST	(((person centred OR Patient centred OR People centred AND chronic long-term conditions OR Multiple chronic illness OR co-morbidities OR Multimorbidity AND Health systems OR Healthcare systems AND Primary care OR Primary health care AND LMICs or resource constraints)	May 2022	Abstract, Publication date from 01/01/2010-30/03/2022, English,	1 631
Google scholar	(((person centred OR Patient centred OR People centred AND chronic long-term conditions OR Multiple chronic illness OR co-morbidities OR Multimorbidity AND Health systems OR Healthcare systems AND Primary care OR Primary health care AND LMICs or resource constraints)	May 2022	Abstract, Publication date from 01/01/2010-30/03/2022, English,	1 820
PubMed	(((person centred OR Patient centred OR People centred AND chronic long-term conditions OR Multiple chronic illness OR co-morbidities OR Multimorbidity AND Health systems OR Healthcare systems AND Primary care OR Primary health care AND LMICs or resource constraints)	May 2022	Abstract, Publication date from 01/01/2010-30/03/2022, English,	660

ABSCOHOST<br><br>Academic search - ultimate<br><br>Academic search complete<br><br>MEDLINE<br><br>Health source (nursing Academic edition)<br><br>Health source – consumer edition	(((Multimorbidity) OR (Multiple chronic long-term illness) OR (co-morbidities) AND person centred OR patient centred) AND (LMICs) AND (Health systems) and (primary healthcare)	May 2022	Abstract, Publication date from 01/01/2010-30/09/2022, English,	4 411
Google scholar	(((Multimorbidity) OR (Multiple chronic long-term illness) OR (co-morbidities) AND person centred OR patient centred) AND (LMICs) AND (Health systems) and (primary healthcare)	May 2022	Abstract, Publication date from 01/01/2010-30/09/2022, English,	4 410

PubMed	(((Multimorbidity) OR (Multiple chronic long-term illness) OR (co-morbidities) AND person centred OR patient centred) AND (LMICs) AND (Health systems) and (primary healthcare)	May 2022	Abstract, Publication date from 01/01/2010-30/09/2022, English,	1
Google scholar	(((Multimorbidity) OR (Multiple chronic long-term illness) OR (co-morbidities) AND person centred OR patient centred OR People centred) AND (LMICs) AND (Health systems) and (primary healthcare)	May 2022	Abstract, Publication date from 01/01/2010-30/09/2022, English,	3 970
PubMed	(((Multimorbidity) OR (Multiple chronic long-term illness) OR (co-morbidities) AND person centred OR patient centred OR People centred) AND (LMICs) AND (Health systems) and (primary healthcare)	June 2022	Abstract, Publication date from 01/01/2010-30/09/2022, English,	1
EBSCOHOST	(((person centred OR people centred OR patient centred AND chronic long-term condition OR comorbidity OR Multimorbidity AND primary health care AND Low- and Middle-income countries AND healthcare systems)	June 2022	Abstract, Publication date from 01/01/2010-30/09/2022, English,	4 519
EBSCOHOST	(((person centred OR people centred OR patient centred AND multiple chronic long-term condition OR comorbidity OR Multimorbidity AND primary health care AND Low- and Middle-income countries AND healthcare systems	June 2022	Abstract, Publication date from 01/01/2010-30/09/2022, English,	6 432
Google scholar	(((person centred OR people centred OR patient centred AND multiple chronic long-term condition OR comorbidity OR Multimorbidity AND primary health care AND Low- and Middle-income countries AND healthcare systems	June 2022	Abstract, Publication date from 01/01/2010-30/09/2022, English,	16 800

PubMed	(((person centred OR people centred OR patient centred AND multiple chronic long-term condition OR comorbidity OR Multimorbidity AND primary health care AND Low- and Middle-income countries AND healthcare systems	June 2022	Abstract, Publication date from 01/01/2010-30/09/2022, English,	12
EBSCOHOST<br><br>Academic search - ultimate<br><br>Academic search complete<br><br>MEDLINE<br><br>Health source (nursing Academic edition)<br><br>Health source – consumer edition	AB (diabetes type 2 or diabetes mellitus type 2 or diabetes 2) AND AB (hiv or aids or acquired human immunodeficiency syndrome or human immunodeficiency virus) AND AB (intervention or programs or implementation or research) AND (afghanistan OR albania OR algeria OR american samoa OR angola OR antigua and barbuda OR argentina OR armenia OR aruba OR azerbaijan OR bahrain OR bangladesh OR barbados OR republic of belarus OR belize OR benin OR bhutan OR bolivia OR bosnia and herzegovina OR botswana OR brazil OR bulgaria OR burkina faso OR burundi OR cabo verde OR cambodia OR cameroon OR central african republic OR chad OR chile OR china OR colombia OR comoros OR democratic republic of the congo OR congo OR costa rica OR cote d'ivoire OR croatia OR cuba OR cyprus OR czech republic OR djibouti OR dominica OR dominican republic OR ecuador OR egypt OR el salvador OR equatorial guinea OR eritrea OR estonia OR swaziland OR ethiopia OR fiji OR gabon OR gambia OR georgia (republic) OR ghana OR gibraltar OR greece OR grenada OR guam OR guatemala OR guinea OR guinea bissau OR guyana OR haiti OR honduras OR hungary OR india OR indonesia OR iran OR iraq OR jamaica OR jordan OR kazakhstan OR kenya OR democratic people's republic of korea OR republic of korea OR kosovo OR kyrgyzstan OR laos OR latvia OR lebanon OR lesotho OR liberia OR libya OR lithuania OR macau OR republic of north macedonia OR madagascar OR malawi OR malaysia OR indian ocean islands OR mali OR malta OR micronesia OR palau OR mauritania OR mauritius OR mexico OR moldova OR mongolia OR montenegro OR morocco OR mozambique OR myanmar OR namibia OR nepal OR netherlands antilles OR nicaragua OR niger OR nigeria OR oman OR pakistan OR panama OR papua new guinea OR paraguay OR peru OR philippines OR poland OR portugal OR puerto rico OR romania OR russia OR rwanda OR samoa OR sao tome and principe OR saudi arabia OR senegal OR serbia OR seychelles OR sierra leone OR slovakia OR slovenia OR melanesia OR somalia OR south africa OR south sudan OR sri lanka OR saint kitts and nevis OR saint lucia OR saint vincent and the grenadines OR sudan OR suriname OR syria OR tajikistan OR tanzania OR thailand OR timor leste OR togo OR tonga OR trinidad and tobago OR tunisia OR turkey OR turkmenistan OR uganda OR ukraine OR uruguay OR uzbekistan OR vanuatu OR venezuela OR vietnam OR middle east OR yemen OR yugoslavia OR zambia OR zimbabwe OR africa south of the sahara OR africa, central OR africa, northern OR africa, southern OR	September 2022	Abstract, Publication date from 01/01/2010-30/09/2022, English,	181

	africa, eastern OR africa, western OR west indies OR indian ocean islands OR caribbean region OR central america OR latin america OR south america OR asia, central OR asia, northern OR asia, southeastern OR asia, western OR europe, eastern OR developing countries)			
PubMed	person centred OR people centred OR patient centred OR differentiated care OR complex care OR Integrated care OR guided care OR collaborative care OR chronic care AND multiple chronic conditions OR multiple long term conditions OR multiple illness OR Non communicable diseases Or complex conditions OR HIV OR AIDS OR Chronic Obstructive Pulmonary Disease OR cardiovascular disease OR Asthma OR diabetes OR hypertension OR stroke OR depression OR anxiety OR chronic arthritis AND primary health care or primary care AND Health systems OR healthcare system AND (intervention or programs or implementation or research) AND (afghanistan OR albania OR algeria OR american samoa OR angola OR antigua and barbuda OR argentina OR armenia OR aruba OR azerbaijan OR bahrain OR bangladesh OR barbados OR republic of belarus OR belize OR benin OR bhutan OR bolivia OR bosnia and herzegovina OR botswana OR brazil OR bulgaria OR burkina faso OR burundi OR cabo verde OR cambodia OR cameroon OR central african republic OR chad OR chile OR china OR colombia OR comoros OR democratic republic of the congo OR congo OR costa rica OR cote d'ivoire OR croatia OR cuba OR cyprus OR czech republic OR djibouti OR dominica OR dominican republic OR ecuador OR egypt OR el salvador OR equatorial guinea OR eritrea OR estonia OR swaziland OR ethiopia OR fiji OR gabon OR gambia OR georgia (republic) OR ghana OR gibraltar OR greece OR grenada OR guam OR guatemala OR guinea OR guinea bissau OR guyana OR haiti OR honduras OR hungary OR india OR indonesia OR iran OR iraq OR jamaica OR jordan OR kazakhstan OR kenya OR democratic people's republic of korea OR republic of korea OR kosovo OR kyrgyzstan OR laos OR latvia OR lebanon OR lesotho OR liberia OR libya OR lithuania OR macau OR republic of north macedonia OR madagascar OR malawi OR malaysia OR indian ocean islands OR mali OR malta OR micronesia OR palau OR mauritania OR mauritius OR mexico OR moldova OR mongolia OR montenegro OR morocco OR mozambique OR myanmar OR namibia OR nepal OR netherlands antilles OR nicaragua OR niger OR nigeria OR oman OR pakistan OR panama OR papua new guinea OR paraguay OR peru OR philippines OR poland OR portugal OR puerto rico OR romania OR russia OR rwanda OR samoa OR sao tome and principe OR saudi arabia OR senegal OR serbia OR seychelles OR sierra leone OR slovakia OR slovenia OR melanesia OR somalia	July 2024	Abstract, Publication date from 01/01/2010-31/12/2023, English,	1666

	OR south africa OR south sudan OR sri lanka OR saint kitts and nevis OR saint lucia OR saint vincent and the grenadines OR sudan OR suriname OR syria OR tajikistan OR tanzania OR thailand OR timor leste OR togo OR tonga OR trinidad and tobago OR tunisia OR turkey OR turkmenistan OR uganda OR ukraine OR uruguay OR uzbekistan OR vanuatu OR venezuela OR vietnam OR middle east OR yemen OR yugoslavia OR zambia OR zimbabwe OR africa south of the sahara OR africa, central OR africa, northern OR africa, southern OR africa, eastern OR africa, western OR west indies OR indian ocean islands OR caribbean region OR central america OR latin america OR south america OR asia, central OR asia, northern OR asia, southeastern OR asia, western OR europe, eastern OR developing countries)			
EBSCOHOST<br><br>Academic search - ultimate<br><br>Academic search complete<br><br>MEDLINE<br><br>Health source (nursing Academic edition)<br><br>Health source – consumer edition	person centred OR people centred OR patient centred OR differentiated care OR complex care OR Integrated care OR guided care OR collaborative care OR chronic care AND multiple chronic conditions OR multiple long term conditions OR multiple illness OR Non communicable diseases Or complex conditions OR HIV OR AIDS OR Chronic Obstructive Pulmonary Disease OR cardiovascular disease OR Asthma OR diabetes OR hypertension OR stroke OR depression OR anxiety OR chronic arthritis AND primary health care or primary care or public health care or community care AND health system or health services or healthcare system AND low and middle income countries or developing countries orafghanistan OR albania OR algeria OR american samoa OR angola OR antigua and barbuda OR argentina OR armenia OR aruba OR azerbaijan OR bahrain OR bangladesh OR barbados OR republic of belarus OR belize OR benin OR bhutan OR bolivia OR bosnia and herzegovina OR botswana OR brazil OR bulgaria OR burkina faso OR burundi OR cabo verde OR cambodia OR cameroon OR central african republic OR chad OR chile OR china OR colombia OR comoros OR democratic republic of the congo OR congo OR costa rica OR cote d'ivoire OR croatia OR cuba OR cyprus OR czech republic OR djibouti OR dominica OR dominican republic OR ecuador OR egypt OR el salvador OR equatorial guinea OR eritrea OR estonia OR swaziland OR ethiopia OR fiji OR gabon OR gambia OR georgia (republic) OR ghana OR gibraltar OR greece OR grenada OR guam OR guatemala OR guinea OR guinea bissau OR guyana OR haiti OR honduras OR hungary OR india OR indonesia OR iran OR iraq OR jamaica OR jordan OR kazakhstan OR kenya OR democratic people's republic of korea OR republic of korea OR kosovo OR kyrgyzstan OR laos OR latvia OR lebanon OR lesotho OR liberia OR libya OR lithuania OR macau OR republic of north macedonia OR madagascar OR malawi OR malaysia OR indian ocean islands OR mali OR malta OR micronesia OR palau OR mauritania OR mauritius OR mexico OR moldova OR mongolia OR montenegro OR morocco OR mozambique OR myanmar OR namibia OR	July 2024	Abstract, Publication date from 01/01/2010-31/12/2023, English,	1654

	nepal OR netherlands antilles OR nicaragua OR niger OR nigeria OR oman OR pakistan OR panama OR papua new guinea OR paraguay OR peru OR philippines OR poland OR portugal OR puerto rico OR romania OR russia OR rwanda OR samoa OR sao tome and principe OR saudi arabia OR senegal OR serbia OR seychelles OR sierra leone OR slovakia OR slovenia OR melanesia OR somalia OR south africa OR south sudan OR sri lanka OR saint kitts and nevis OR saint lucia OR saint vincent and the grenadines OR sudan OR suriname OR syria OR tajikistan OR tanzania OR thailand OR timor leste OR togo OR tonga OR trinidad and tobago OR tunisia OR turkey OR turkmenistan OR uganda OR ukraine OR uruguay OR uzbekistan OR vanuatu OR venezuela OR vietnam OR middle east OR yemen OR yugoslavia OR zambia OR zimbabwe OR africa south of the sahara OR africa, central OR africa, northern OR africa, southern OR africa, eastern OR africa, western OR west indies OR indian ocean islands OR caribbean region OR central america OR latin america OR south america OR asia, central OR asia, northern OR asia, southeastern OR asia, western OR europe, eastern			

Online Supplemental Table 2: **Bibliometric information, methodological characteristics, and key findings of reviewed studies n=36).**

First author and Year of publication	Country/ study setting	Aim/purpose	Study design	Population and sample size	Barriers	Facilitators/Needs
Juma Kenneth, 2018	Sub-Saharan Africa	To synthesize published literature on noncommunicable disease behaviour, change communication interventions in sub-Saharan Africa among persons living with HIV in the general population and to inform efforts to adopt similar HIV and NCD BCC intervention activities.	Narrative review	20 articles in the Sub-Saharan Africa and 10 Key informants.		Health education (dietary issues, exercising, drug intake etc), Community-based programs to empower patients. Social marketing (radio, television, internet, billboards etc), Marketing healthy lifestyle to parents of school-going children. Motivational interviewing, Mobile health (m-Telemedicine, m-Learning) to improve service uptake and behaviour modification. Peer support to enhance self-care behaviour. Community outreach and Mass media. Use of social role models and opinion leaders to deliver interpersonal messages. More quality research to scale up health promotion interventions.
Balcha T. Taye, 2015	Ethiopia	To compare the outcome of anti-tuberculosis treatment in HIV-positive adults diagnosed with TB through active case-	Prospective cohort studies	439 TB/HIV co-infected participants	Late diagnosis of active TB cases. Increased transmission and death of carriers. Lack of effective diagnostic algorithms and tools for TB detection in people living with HIV. Low adherence to drugs.	Active case finding procedures to detect TB cases. Improved survival, Reduced risk of TB transmission. Outreach mechanisms using mobile clinics enhanced reach to the community. Collaborative TB/HIV interventions. Use of modern TB diagnostic tests prior to ART initiation.

		finding or passive case-finding.				
Mohr Erika, 2017	South Africa	To assess the impact of self-administered treatment in a South African township with high rates of rifampicin-resistant tuberculosis and HIV	Non-Randomised Comparison	174 TB patients who completed the intensive phase of self-administered treatment.	Ineffective treatment options. Stigma due to facility-based DOTS. Clinical resource constraints. Treatment administration hinderance. Gender discrimination. Long distances from the clinics. Substance abuse.	Collaboration of partners enhanced healthcare service delivery within the community. Decentralisation of the people-centred care model. Implementation of adherence-support strategies. Health education. Community-based adherence support. Patient support provisions to enhance adherence. Community Health Workers Support services.
Matenge G. Tjedza, 2018	Botswana	This study aimed to explore the barriers to accessing cervical cancer screening in women with HIV in Kgatleng district, Botswana.	Phenomenological qualitative study.	14 HIV positive women	Weak primary care system. Insufficient health promotion. Poor communication skills. Poverty. Poor access to health services. Low utilisation of cervical cancer screening services. Occupational factors and biological variables. Cultural beliefs and values distracted biomedical treatment efforts. Long distance from PHC facilities. Unpredictability of public transport. limited access to PHC facilities. Poor working conditions. Inefficient services. Inadequate equipment performance. No clinical guidelines. Inefficient services delivery. Lack of person-centred communication. General lack of knowledge about screening and cervical cancer in general. Negative attitudes from clinic staff. Poor communication pathways of results. Shortage of nurses for accessing	Increased awareness and motivation for service uptake. Improved and TB health education for HIV. Effective integration of health education on cervical cancer into services for HIV users. Embracing alternative and emerging technology. Intensifying appointment and referral systems. Behaviour changes counselling. Reduced alcohol and substance abuse. Enhanced community-oriented health education.

					cervical smears. Inadequate health promotion. Lack of stakeholder engagement. Power uptake of health promotion services. Lack of confidence in services.	
Malan Zelra, 2015	South Africa	The study reports on a qualitative sub-study, which explored whether a training intervention changed Primary Care Providers' perception of their confidence in their ability to offer brief behaviour change counselling, whether they believed that the new approach could overcome the barriers to implementation in clinical practice and be sustained, and their recommendations on future training and integration of brief behaviour change counselling into curricula and clinical practice.	Qualitative study	23 nurses in their diploma level 1, 12 family medicine registers during their second year of training, 2 general practitioners in private practice.	Fewer resources. Poorly prepared services for chronic care. Inadequate training in counselling skills. Language barriers. Poor knowledge of lifestyle modification. Poor continuity of care. Doctors have limited time for counselling. Lack of adherence to drugs. Few staff to offer organisational support for future training. Lack of support from within the facility. Poor care continuity. Significant misalignment of personal and organisational values. Organisational culture is not aligned to patient-centred care guidelines. Unsupportive and undermining environment. Weak health systems often make it difficult to implement and sustain quality service interventions. Unsupportive organisational culture. Lack of educational resources.	Behaviour changes, counselling programs. Health professionals more confident in their ability to help patients with behaviour changes. Embrace nurses to provide counselling services as part of integrated management of chronic conditions. Incorporate behaviour change counselling as part of HWCs training into undergraduate curricular as well as part of continuous training of PHC workers. Need for leadership transformation to enable people-centred care needs.
Murphy Katherine, 2015	South Africa	The aim of the study was to explore patients' current experiences of chronic care, as well as	Qualitative	22 hypertensive and diabetic individuals attending three	Doctors not well knowledgeable on person-centred care. Negative attitudes of healthcare providers. Unprofessional contact by nurses. Poor patient	Lifestyle modification and adherence to medication programs. Healthcare providers empowered the users with a sense of control and choice over health-

		their motivation and capacity for self-management and lifestyle change.	interviews	public sector community health centres.	engagements strategies by nurses. No shared decision-making opportunities, about treatment, self-management, and lifestyle modification. A general lack of education materials and adequate health information. Lack of effective counselling and social support systems. Lack of appropriate communication skills by HCWs. Lack of well-defined continuity of care mechanisms.	related decisions. Users reported feeling more comfortable discussing their concerns about self-management with community health workers. The self-management support systems enhanced users' knowledge, motivation skills of behaviour change and empower users to make choices about treatment options. Practical guidelines about lifestyle modification were provided. Building collaborative and supportive relationships between service users and HCWs. Realignment of policy frameworks, training programs, research to patient centred care models improved service delivery.
Gupta-Wright Ankur, 2019	Sub-Saharan Africa	Exploration of HIV and TB diagnostics that could be used at point-of-care in any settings and outline some important principles and applications of implementation science to aid their application and use.	Implementation science	HIV and TB diagnostics	Expensive equipment. Skilled technicians required. Unreliable electricity and water supplies. Unstable operating environments. Most laboratory tests were inaccessible to patients and clinicians. Lack of diagnostics equipment and supplies.	Embracing implementation science promoted the scaling up and use of evidence-based practices that improved quality and outcomes. Resource optimization enhanced health care efficiency.
Chang Y Angela, 2019	South Africa	This study presented the prevalence of multimorbidity that includes HIV in the complex epidemiological setting of South Africa. It filled a gap in the	Cross-sectional study	5059 people aged 40+	High health expenditures. Frequent service utilisation. Lack of integrated clinical guidelines for multimorbid care. Negative user experiences. Long waiting times. Staff shortages. Poor integration between vertical HIV programmes and general services. No conducive environment for	Promote antiretroviral treatment (ART) services. Awareness campaigns for integrated care services. Provision of long-term integrated care for multimorbidity users. Intensifying awareness campaigns for chronic multimorbidity. Integrating clinical

		multimorbidity literature that is being dominated by studies in high-income or low-HIV prevalence settings.			implementation of the Integrated Chronic Disease Management models.	guidelines into routine provider training enhanced multimorbid care.
Pang Y. Wang, 2019	Brazil	The investigation depicted patterns of multimorbidity in the general population and examined its association with the individual- and area-level factors in an urban sample of non-elderly adults of Brazil.	Cross-sectional	1,571 community-dwelling women for Psychiatric disorders/ Hypertension	Low educational level. Low country's income level. Social inequality. Insufficient budget allocated to the health sector. Persistent disparities in access to health-care services. Poor socio-economic status. Poor uptake of multimorbid services.	Integration and scaling-up of the mental health services into primary care efforts. Re-engineering of the healthcare delivery system. Proper policy planning and forecasting. Enhanced patient-centred care systems. Delivery of early preventive and treatment programs. Replacing the single-disease framework by person-centred models in PHC settings. Readapt and strengthen health systems to be responsive to MLTC-M epidemic in a person-centred holistic approach.
Nkhoma, Kennedy, 2018	Kenya	We aimed to measure the three-day period intensity of problems and concerns (physical, psychological, social and spiritual), and identify predictors of problems and concerns, among HIV patients with other comorbid conditions attending outpatient care.	Cross-sectional self-report quantitative study	HIV/AIDS/TB/ Cancer	Stigma. Lack of quality palliative care services.	Enhanced care continuity to control symptoms associated with TB and comorbidities. Integration of TB and palliative care services. Improved patient-centred care services. Promotion of drug adherence. Training of HCWs on palliative care principles. Enhanced community-based management and support of users.

Hugo Jannie F.M, 2020	South Africa	This article presented the results of an applied research initiative to facilitate the coordination of patient care.	Qualitative study	72 ward rounds conducted weekly at three hospitals.	Lack of functional, interprofessional care- coordination. Lack of integrated coordination subverted essential relationships between users and families in their homes and communities. Poor patient referral contributes to an increased service burden. Bottlenecks and inefficiencies. Lack of care coordination causses HCWs to be overwhelmed by service demand. Hierarchical organisation of systems within PHC systems affects decision-making.	Employing safe, accessible, and efficient quality services. Collaboration of professionals in healthcare service at various levels. Provision of integrated coordination put healthcare plans into practice. Team collaborations from different sectors enhanced service delivery.
Van Rensburg A. Janse, 2020	LMICs	This paper aimed to review the nature and extent of tuberculosis and common mental disorder comorbidity and assess person-centred tuberculosis care in low-to-middle income countries and emerging economies.	Scoping review	100 articles looking at comorbidities between tuberculosis and common mental disorders.	Stigma in relation to mental illness and TB. TB treatment non-adherence. Alcohol abuse caused neglect of TB treatments. Severe physiological reactions. Financial and psychological challenges. Lower socioeconomic backgrounds. Lower education backgrounds. Financial and social strains. Increased expenditure in accessing care. Removal from social networks. Loneliness and medication side effects. Poverty leading to increased alcohol abuse. Downward social mobility. Fragmented, non-dependable health information systems. Limited patient management. Fractured referral and communication between and within levels of care. Lack of standardisation and fragmentation in the use of different concepts. Gender	Developing interventions collaboratively assisted in strengthening stakeholder capabilities, opportunities, and motivations, resulting in more sustained psychological support throughout treatment processes. Enhanced positive stress management skills. Engaging and empowering family provided emotional support and care. Well-trained social workers supported users in decision-making. Established sustainable linkages among the care-coordinating team. Established referral systems to connect users to a wide range of service providers. Enhanced service coordination promoted continuity of care pathways.

					disparities. Limited education and failing health systems.	
Yakubu Kenneth, 2018	Nigeria	The aim of this study was to explore perceptions of family-centred care among users with chronic diseases.	Mixed-methods phenomenological study	21 adult users with chronic diseases at a general outpatient clinic in north-central Nigeria.	Low health education. Low socio-economic status. Limited family member support systems when needed. No shared decision-making opportunities during consultations.	Enhanced health education programs. Empowered family members promoted family-centred care. Improved financial and psychosocial support from families. Modifying health seeking behaviour from families. Encouraging drug adherence.
Naidoo Keshena, 2019	South Africa	The aim of this study was to explore the experiences and expectations of people aged 60 years and above regarding ageing and health services, and the factors that might improve the quality of primary care services for geriatric patients.	Qualitative study	28 participants aged 60 years and above.	Lack of sufficient data on the health needs of geriatrics in SSA to inform policy and practice. Financial constraints for healthcare cost and transport. Limited access to facilities. Geriatrics with physical impairments and urinary incontinence were discouraged to attend health facilities with long queues. Specialised geriatric services and specialist geriatricians were rarely available. Poor coordination of care. Polypharmacy in geriatric patients led to non-adherence to drugs. Long waiting times. Lack of caring from professionals. Pill burden. Shortages of medication. Lack of coordination of care. Professionals were perceived to view elderly patients as diseases to be treated rather than individuals with health needs. Lack of interest in patients' concerns. Poor	Modified primary care services improved the quality of care for the elderly. Give greater importance to promotive and preventive care. Enhanced integration of various existing services. Continuous training of health care workers. Improved exposure to geriatric medicine.

					understanding of medication purpose. Little information or education about prescribed treatment. Multiple adverse and drug effects. Fragmentation of services and multiple concurrent medications prescribed. Transport cost burden for return visits to collect medications that were out of stock.	
Rzewuska Magdalena, 2020	Brazil	The aim of this study was to advance the understanding of complexity inherent to treatment adherence in mental-physical multimorbidity and identify opportunities for specific solutions to improve adherence in Brazil.	Qualitative study	80 people with 18 years and above who had mental-physical mental multimorbidity in 5 sub-groups	Multiple concurrent prescriptions causing non-adherence. Low health literacy. Polypharmacy. Lack pf time and financial resources. Inadequate space at PHC facilities. Skipping or missing doses (taking medicine at irregular intervals. Negative social influences to resist medication. Poorly coordinated or fragmented care. Limited exchange of communication.	Medicines subsidized by the government. Patient follow-ups, Health education. Home visits and referrals to secondary mental and physical health services and social services. Training in Patient Centred Medicine. Shared decision-making. Enhancing the patient-clinician relationship. Promoting Integrated care approaches. Enhance patient-centred care. incorporating patient priorities into treatment plans. Lifestyle modification. Enhancing involvement of patients in decision making. Providing practical support to mitigate financial barriers. Enhance treatment adherence mechanisms. Promote continuous contact between health professionals and the patients. Enhanced continuity of care. Effective health information systems. Prioritise motivation-matched-person centred treatment goals.
Amu Hubert, 2021	Ghana	The study was aimed at exploring the practices and challenges associated with the management of chronic	Qualitative study	82 chronic non-communicable diseases patients	Late presentation of illnesses at PHC services. Language barrier between service providers and users. Work-related stress stemming from heavy workloads. Poor utility supply.	Mosques, churches, and other religious institutions, for instance, played essential roles in the spiritual actions people take in managing their chronic conditions. National health policies support the

		no-communicable diseases by patients and health professionals.		and 30 health professionals.	Inadequate logistical mechanisms. Lack of staff motivation. Poor infrastructure and equipment. Lack of in-service training. Inadequate equipment hindered chronic care and management. Poor working conditions for health workers demotivated them. Low number of health professionals. No training for emerging technologies and strategies for chronic care. Poor health literacy level. Financial challenges of users. Unaffordable medications. Some medications not covered by the country's social health insurance policy. Users not able to purchase anthropometric equipment for monitoring their health at home. Some users not able to go for their reviews on time due to financial constraints. Non-adherence to treatment schedules. Users mixing herbal medications with the medical ones. Users replaced the biomedical medications with herbal medications completely. High poverty rate. Inadequate political commitment by states to accelerate chronic care priorities in the national development agenda.	prevention and control of chronic. Enhanced PHC services and clinical care including early detection. Training of health care professionals and patients. Promoting self-management support. Development of health professional capacity. Health professionals empowering users on the need to take their medications. Proper storage of medications. Patients used anthropometric equipment to monitor their health status at home. Developed or expanded innovative policy interventions improved chronic care. Availability of evidence-based health care workers education toolkit. Promoting clinical care consistent with evidence-based guidelines.
Kamvura, Tiny Tinashe, 2021	Zimbabwe	The study used a theory of change approach to formulating a road map on how to successfully integrate diabetes and hypertension care into	Theory of change, qualitative study.	18 stakeholders from diverse backgrounds included grandmothers working on the	Low detection and control rates for diabetes and hypertension. Vertically organised care systems. Multiple consultation days for a single user. Multiple clinic visits for users. Chronic condition services were chronically	Availability of essential diagnostic tools and medications at PHC facilities. Using standardised protocols and calibrated equipment for diagnosis, treatment, monitoring. Improved referral pathways to specialist care. Enhanced task-shifting

		the existing Friendship Bench to develop an integrated care package for depression, hypertension and diabetes aimed at strengthening non-communicable disease care in primary health care systems in Zimbabwe.		Friendship Bench project (n = 4), policymakers from the ministry of health (n = 2), people with lived experience for the three NCDs (n = 4), health care workers (n = 2), and traditional healers (n = 2).	underfunded. Massive shortage of trained healthcare practitioners. Low health literacy. Communities had low awareness of preventive measures for hypertension and diabetes. Grandmothers overburdened with workloads. High mobile data costs on implementing proposed interventions.	models using trained community grandmothers. Adapted existing evidence-based interventions to deliver person centred care. Effective stakeholders' engagement. Enhancing community support groups. Using WhatsApp platform to access important communication and dissemination of knowledge to improve referrals pathways. Social media use strengthened NCDs services provision in the face of the COVID-19 pandemic. Community health workers use was cost-effective. Community health workers were trained in providing counselling services to primary care users. Integrated care package alleviated burden on HCWs. Embracing mHealth and m-Learning. Improving access to person centred care. Enhanced referral pathways and appointment systems. Improved health seeking behaviour. Enhanced self-management support. Integrated multicomponent interventions to address risk factors.
Masuma Pervin Mishu1, 2021	LMICs	To assess the effects of pharmacological, behaviour change, and organisational interventions versus active and non-active comparators in the prevention or delay of type 2 diabetes among	Literatu re review	30 studies included.		Enhanced research efforts on behaviour change and organisational interventions. Applying health behaviour change theories such as social cognitive theory, the theory of reasoned action and planned behaviour. Improved health promotion efforts. Improved self-management interventions. Enhanced capacity building and training programs to

		people with mental illness in LMICs.				improve communication and skills on disease conditions.
Godongwana Motlatso, 2021	South Africa	This study investigated the challenges faced by health care providers in delivering the outcomes of the Integrated Chronic Disease Model, particularly, to users living with the comorbidity of HIV and hypertension or diabetes as well as providing the perspectives of persons living with these conditions.	Phenomenological qualitative study.	12 health care providers and 12 adult participants with HIV/AIDS, hypertension, and diabetes.	Under-resourced facilities. Inadequate staff capacity. Nurses frequently overwhelmed with the users' flow. lack of training and guidelines on co-occurring chronic conditions service delivery. Lack of integrated care training. Some staff could not find time or financial resources for training. Multiple appointment system for different conditions. Stigma and discrimination caused non-disclosure. Polypharmacy (the use of multiple medications potentially resulting in treatment burden, side effects). Poor knowledge of treatments. Constant movement of service users. Low socio-economic status. Poverty. Lack of social services such as counselling and psychosocial support. Poor access to healthcare facilities. Challenges in maintaining a healthy diet. Poor health education. No support groups and adherence clubs in communities.	Providing capacity building and training to improve the delivery of chronic care treatment. Assisted self-management. strengthening activities for comorbidity health promotion. Embracing integrated care models to improve care outcomes of different chronic conditions. Enhanced health promotion strategies. Improved health education. Embrace social medial platforms (WhatsApp) for efficient information dissemination. Establish adherence clubs to support one another. Enhanced partnerships. Better management of the allocation and distribution of resources. Providing a well-monitored integrated care model.
Thornicroft Graham, 2018	LMICs	The review described the most relevant concepts and models of integrated care for people with chronic (or	Review study.	18 research papers and 3 book chapters on Depression, cardiovascular		Collaboration of partners enhanced healthcare service delivery within the community. Decentralisation of the people-centred care services improved coverage. Implementation of adherence-

		recurring) mental illness and comorbid physical health conditions, providing a conceptual overview and a narrative review of the strength of the evidence base for these models in high-income countries and in low-income and middle-income countries, and identified opportunities to test the feasibility and effects of such integrated care models.		disease, TB/HIV.		support strategies. Patient encouragement. Community-based adherence support. Empowered users would take responsibility for their own treatment adherence. Enhance patient centred care approaches. Improved access to PHC services. Promoting users access to services. Adapt lessons learnt in previous programs into current ones.

Barua Kabita , 2018	India	The aim of the study was to assess morbidity pattern and health-seeking behaviour in two urban slums in India and to understand the role of Vitamins in health seeking.	Cross section al study	A total of 1025 households representing 4997 family members with hypertension, diabetes, HIV, Tuberculosis, asthma, mental disorders, cardiovascular diseases, cancer were surveyed.	High illiterate level. Low level of education. Poor awareness of health problems. Poor socio-economic status.	Expanded scope of PHC services. Integrating adequate primary curative care services to multiple noncommunicable diseases. Increased access of people of poor economic status to health care facilities. Reduced cost of health expenses. Enhanced referrals and follow-up mechanisms for users suffering from chronic noncommunicable diseases.
Uwimana Jeannine, 2012	South Africa	The study assessed the level of non-governmental organisations and community care workers' engagement in collaborative TB, HIV and Prevention of Mother to Child Transmission (PMCTC) activities and or explored how CCWs could provide integrated TB/HIV/PMTCT care at the community level. South Africa,	Mixed method study	37 facility managers, 32 NGOs managing HBCs,1 NGO managing CHWs, 3,867 household members randomly selected from the catchment areas of audited facilities and NGOs, 29 Provincial managers – 6 District managers – 6 Facility	Poor governance structures. Inadequate skills of CCWs. Insufficient financing for community-based activities. Lack of effective structured linkages of key sectors. Inadequate supervision of CCWs. No formal structural mechanism that links NGOs-CCWs with PHC clinics. Shift of leadership and supervisory accountability. Lack of the needed training for CCWs and deficiency in essential skills. Most CCWs were trained according to the funders' agendas and not to existing healthcare needs. Disease-based and verticalized care systems were prevalent. Lack of comprehensively trained packages for CCWs. Lack of transport for CCW supervisors to cover sparsely populated districts. Lack of recognition of CCWs as state	Enhanced linkages between facilities and NGOs. Embracing training provided to community care workers CCWs. Emphasized home based care training. Promoting disease screening at community level. Integration of treatment services to reduce multiple visits to PHC facilities saved both time and money. Development of proper supervisory mechanisms for CCWs was crucial. Trained HCWs provided multiple services (multi-tasking). CCWs expanded their scope of practice to include person-centred multimorbid care services. Development of a proper monitoring and evaluation framework to track community-based activities. Enhance multistakeholder collaborations.

				managers – 11 NGO managers- 6 FGDs with CCW- 4 FGDs with patients- 2.	employees and part of the formal health system. Poor relationships between CCWs and health care workers. HIV stigma in the community promoted lack of disclosure.	Increased opportunities for community and facility interfaces.
Tapia-Conyer Roberto, 2016	Mexico	The paper focused on finding and assessing relevant enablers (elements that allow and promoted the rapid and successful implementation of the Casud Model) and inhibitors (elements that might impede or hinder the said implementation process).	Mixed methods study	NCDs/Type diabetes		Political support for the Casalud model. The Casalud Casalud is a comprehensive primary healthcare model that was implemented in Mexico to enable proactive prevention and disease management throughout the continuum of care, using innovative technologies and a patient-centred approach. Its care elements were made to improve care quality by standardizing healthcare protocols and implementing continuous monitoring.
Chua S. Siang, 2012	Malaysia	This study aimed to identify the types of pharmaceutical care issues encountered by primary care users with diabetes mellitus, hypertension or hyperlipidaemia in Malaysia.	Qualitative controlled trial	477 participants with diabetes mellitus, hypertension and hyperlipidaemia in primary care settings.	Non-adherence to medications due to drug abuse problem. Incorrect administration of doses and frequencies or incorrect timing with respect to meals. Adverse drug reactions (ADRs) included side effects of medications. Non-adherence to medications was attributed to drug abuse problems and lack of confidence in the healthcare system. Users were not loyal to medication prescriptions due to fear of side effects.	Enhanced counselling of users on medications adherence. Proper referral of users to the prescribers. Educating users concerning their disease states (Health education). Education on the dose or frequency of the medications. Constant monitoring of patients' condition. Provision of pharmaceutical care. Collaboration of health care workers. Enhanced health promotion interventions.

Rachlis Beth, 2016.	Kenya	The study aimed to explore community perceptions of community health workers including perceptions of their roles in chronic disease management as part of the Academic Model Providing Access to Healthcare Program in western Kenya.	Qualitative research	16 in-depth interviews with religious leaders, traditional healers, primary healthcare workers, care ministry workers and village elders.	Lack of trust in CHWs inhibit their ability to effectively deliver on their mandate. Lack of professional adequacy and skills mix for CHWs. Lack of adequate health information on all the relevant diseases. Lack of health information among CHWs stemmed from little education and/or a lack of formal training. Lack of positive and sustainable ways for patients to manage their own health. Individuals too reliant on monetary incentives and not fully in charge of their health.	Encouraging awareness about diseases (HIV and TB) and prevention and treatment. Encouraging health education programs. Use of religious Leaders to advocate for health living increased uptake of services. CHWs contributing to service provision, facility linkage and de-stigmatization. Community members had trust and confidence with CHWs' conduct. CHWs worked as role models in their community. CHWs are perfectly positioned to bridge a gap between people and facilities. CHWs increased knowledge and support to promote continuity of care among users. The use of social gatherings, community gatherings, schools, churches, youth groups to disseminate important health information was helpful.
Peer Nasheeta, 2020.	South Africa	The aim of this study was to evaluate the perceptions and experiences of people living with HIV infection and comorbid hypertension, and their healthcare providers, related to their diagnoses and interactions with chronic healthcare services in South Africa.	Mixed methods study	Interviews were conducted with clinicians (n = 11), specialised nursing professionals (n = 10), lay counsellors (n = 12), six patients focus groups (n = 35) and 20 in-depth individual patient interviews. With	High pill burden. Lack of continuity of care received. Patients seen by multiple doctors who diagnosed them with different conditions each time they visited the PHC facility. Incompetent doctors. Users developed anxiety and uncertainty due to multiple prescriptions. Insufficient healthcare providers contributed to the long waiting times. Long waiting times were a barrier to optimal patient care. This in turn affected adherence to drugs. Greater defaults on scheduled appointments.	Integration of services. Advocating against stigma and discrimination improved service uptake. Specialised training in integrated care management. Dedicated care teams for multiple chronic conditions.

				HIV and Hypertension.		
Pati Manoj Kumar, 2020	India	This paper aimed to analyse existing gaps in the organization and integration of NCD services at primary care and suggesting plausible solutions that existed.	Narrative review	NCDs	CHWs had limited capacity to make effective follow-up of users. CHWs lacked constant supportive supervision. Lack of health promotion guidelines leading to non-adherence to treatment and poor service uptake. Limited number of health professionals reduced NCDs screening capacity. Inefficient referral system. Poor patient recording system. Poor implementation strategies for emerging chronic care programmes.	Adequate facility readiness provided an enabling environment for high quality of care. Leveraging existing lessons enabled implementation of best practices on emerging programmes. Development of web-enabled strategic management information systems improved programme management and monitoring situations. Expanded investment in implementation research of multimorbidity. Integration of NCDs care in primary health care services. Capacity building for the existing HCWs. Early disease diagnostics, and screening of risk factors. Enhanced understanding of disease burden at population level. Development of monitoring indicators, and an information system for effective programmatic decision-making. Embracing specific global models to inform technological policy. Embracing innovative measures in the health care system for screening, diagnosis, and management.
Kamvura T Tinashe Kamvura, 2022	Zimbabwe	The study aimed to explore perceived barriers to providing non-communicable	Qualitative	10 participants with diabetes, hypertension, and depression	Medication shortages for non-communicable conditions. The shortage of equipment for screening and testing purposes. Clinic with no essential	Improved health investment for NCDs' prevention led to better health outcomes with minimum funding for resource-constrained healthcare systems. Task-

		diseases care in primary health care facilities in Zimbabwe.	researc h	from five busy urban clinics.	resources needed by patients would refer to private facilities which were more expensive than public PHC care. High cost of alternative services presented a challenge for the users.	shifting care for chronic conditions. Continual lobbying policymakers to priorities person centred care. Enhancing multisectoral approach. Improving working conditions such as basic clinical supplies and essential drugs. Initiating and maintaining screening at the community level through CHW services. Reducing work burden on nursing staff.
Jinda Devraj, 2018.	India	The study aimed to describe the steps and processes in the development of mobile WellCare, a complex intervention based on mobile health technology.	Cluster ed random ised trial	A total of 631 patients diagnosed with hypertension and/or diabetes were included.	Inadequate health professional capacity and resources. Lack of training for health care providers to effectively perform their duties. Limited access to evidence-based guidelines for the integrated management of hypertension and diabetes. Limited knowledge update regarding management of diabetes and hypertension. Lack of an organized system for patient screening, registration, record keeping, long-term follow-ups, monitoring of process indicators. Limited drug supply and diagnostics for hypertension and diabetes management. Limited evidence to inform interventions and their implementation.	Consultations to develop integrated chronic diseases management approaches were essential. Enhanced use of m-Health applications. Incorporating several change-management strategies including training and orientation of CHC healthcare team on the mHealth application. Regular monitoring of the delivery of intervention through both m-health applications data and in-person mechanisms.
Clouse Kate, 2017	South Africa	The study sought to explore healthcare utilization among postpartum women requiring chronic management of HIV	Qualita tive researc h	25 women who were 18years and above, at the Developmental Pathways for Health Research	Long waiting period. Poor and misplaced paperwork. Disrespectful treatment by clinical staff/lack of professionalism. Lack of integration of HIV and NCDs treatment services. High healthcare utilization. Lack of	Effective social and family support systems enhanced disease management.

		and non-communicable diseases and identify facilitators and barriers to follow-up engagement.		Unit at Chris Hani Baragwanath Academic Hospital in Soweto, South Africa with HIV and diagnosed with an NCD.	treatment adherence. Shortage of financial resources. Limited time for effective patient consultation and counselling. Lack of information of diagnosis outcomes from clinic staff disturbed postpartum visits.	
Mohamed O. Haroon, 2015	South Africa	The aim of this paper was to describe the development and implementation of the integrated chronic disease management model for South Africa.	Qualitative study of assessing the operational efficiency of the Integrated Chronic Disease Model	Chronic infectious diseases and non-communicable diseases.	Verticalized care services were prominent. Erratic provision of chronic care services. Bottlenecks and extended patients' waiting times. Poor appointment systems. No proper patient follow-ups resulted in defaulters. Inadequate HCWs. Inequitable distribution of HWCs across settings and sectors. Staff incompetence. Lack of a competent managerial capacity. Uneven allocation of resources. Inappropriate staff allocation. Poor clinical recording systems. Poor quality of data collecting mechanisms.	Re-engineering of the PHC system. Enhanced teamwork improved service delivery capacity. Proper monitoring and supportive supervision frameworks. Development of a patient-centred appointment system. Integration of patient records. Provision of sufficient rooms for chronic patients' consultations. Enhanced clinical management support for chronic conditions. Capacity building for nurses for integrated care services. Improved clinical information system promoted continuity of care. Assisted self-management support. Empowering users for self-care. Establishment and support of ward-based outreach teams. Scaling efforts for health promotion talks at households. Training of healthcare workers to multi-task. Encouraging task-shifting among lay-workers. Improved medical supply and management system. Equipment supply audit. Integrated data collection tools for all chronic conditions. Enhanced leadership and advocacy mechanisms.

Koros Hillary, 2023	Kenya	This study aimed at understanding the impact and scalability of a novel approach to integrate promotive, preventive, and curative care for diabetes, hypertension, cervical and breast cancer at the primary health care level within the Academic Model Providing Access to Healthcare programme in Western Kenya.	Cross sectional quantitative study	The model of care was implemented across a total of 73 facilities with 300 service users with hypertension and/or diabetes.	Low health education. Low income. Medical and health care expenses beyond the reach of most people. Lack of self-monitoring equipment at home. A higher burden for medical expenses. Limited funds to secure required special diet. Irregular and inefficient medical appointment system. Few people had health insurance cover. Inadequate social support.	
Sansbury M. Griffin, 2023	Malawi	The aim of this study was to identify discrete leadership strategies that facilitate overcoming barriers to the integration of depression care in NCD clinics in Malawi and to understand how clinic leadership shapes the implementation climate.	Qualitative study.	39 participants that included the District Medical Officer, the NCD coordinator, one NCD provider, and a research assistant from each of the 10 study sites.	Insufficient HCWs were overburdened by a heavy workload. Insufficient supply of essential medicines. Lack of motivation for healthcare providers. Authoritarian leadership style caused negative attitude among providers. Leaders not providing platforms for shared decision making with other health care professionals. Lack of mental healthcare initiatives. Low mental health literacy.	Leadership engagement fuelled a positive implementation environment of health care services. Leaders were actively assisting in daily operations of the clinical services and problem-solving. Enhanced teamwork in clinical interventions development increased provider responsibility. Fostering a supportive learning environment by recognising providers efforts. Maintaining effective open lines among leadership structures. Clear treatment guidelines increased knowledge for providers. Enhanced integration of depression treatment programs into existing NCD care models. Mentorship and hands-on learning created a productive implementation climate for healthcare interventions. Leverage

						existing interventions to promote task-shifting services. Leadership-driven service delivery improved task-shifting of integrated PHC services.
Hendricks, Lynn, 2023	South Africa	The purpose of this qualitative study was to explore decision makers' perceptions of developing population-level interventions (policies and programmes), targeting risk factors for hypertension and diabetes, in South Africa.	Qualitative study	12 individual interviews and 1 group interview (consisting of 3 participants).	Lack of supportive policy and program. Limited time for user consultation. Lack of resources to support community-based work. Lack of stakeholder consultation and buy-in during policy formulation. Ineffective user monitoring and evaluation mechanisms. Insufficient funding resources for research to inform health policies. Competing interests between private and public sectors. Lack of modern data monitoring systems. No control on unhealthy foods advertising. Ineffective community-engagement processes. Low computer literacy levels and limited access to internet.	Enhanced community engagement processes. Collaborative process being driven by the policy unit. Intensified research partnerships to inform new policies. Co-creating enabling environments for sustained healthy lifestyle choices. Partnership with tertiary institutions and other seasoned health specialist scaled up quality service delivery. Contextualisation of policies and programs addressed local needs. Provision of an enabling environment that balanced both economic and nutritional needs. Embracing ongoing quality improvement models. Incentivising community implementers and participants.
Summer-Brooke Carrie, 2022	South Africa	The study aimed at qualitatively describing managers' experiences of implementing the MIND programme and their insights into potential strategies for supporting sustained implementation.	Qualitative study	Two FGDs and eight in-depth interviews were conducted with 30-50 years old managers of urban and rural primary care facilities in the	Top-down communication and decision-making made staff resistant to new services. Lack of private spaces for counselling within facilities constrained service delivery.	The need for staff reorientation and up-skilling to improve mental health services. Creating adequate time for quality counselling of patients. Strengthening linkages between the health and social service sectors to facilitate delivery of integrated mental health services. Managers recommended ongoing monitoring of the service and communication about its intervention impacts. Investing in managers' capacity to support effective implementation of

				Western Cape province.		interventions and innovations. Clinical improvements in service users receiving counselling. Improved user awareness to self-care strategies. Reduced alcohol intake improved adherence to chronic medication. Developing clear referral pathways improved care for users. Providing convenient appointment times to eligible patients.

Online Supplemental Table 3: Themes and sub-themes

Pillar	Barriers	Facilitators
**PEOPLE**:<br><br>Health needs and expectations	**Low education level**<br><br>- Poor health literacy<br>- Low service utilisation<br>- Late presentation at health facilities<br>- Poor decision making<br><br>**Stigma and discrimination**<br><br>- Poor health seeking behaviour.<br>- Non-disclosure.<br>- Poor service uptake<br>- Poor adherence to treatment and appointments.<br><br>**Poverty**<br><br>- Inadequate money to buy special diet foods<br>- Inadequate money to procure essential medicines<br>- No transport money for regular visits to health facilities.<br><br>**Substance abuse**<br><br>- Self-neglect<br>- Addiction<br>- Non-drug adherence<br><br>**Alternative sources of care**<br><br>- Traditional and religious healing methods.<br>- Negative user perceptions about medical treatments<br>- Non-drug adherence.	**Collaborations**<br><br>- Provider - service user collaborations<br>- Shared decision-making between service users and providers<br>- Coordinated support pathways between PHC service providers and family members.<br><br>**People and community empowerment**<br><br>- Person-centred health promotional programs<br>- Self-management support<br><br>**Social participation**<br><br>- Community supported programs<br>- Community networks<br>- Adherence clubs<br>- Lifestyle modification programs<br><br>**Shared decision making**<br><br>- Between service providers and users<br>- Respect for service user values and preferences.<br>- Empowers service users to make informed decisions.

**GOVERNANCE:** Intersectoral governance and accountability	**Leadership and policy frameworks**<br>- Limited political commitment to multimorbid care programs.<br>- Health organisational structures not supportive of multimorbid care.<br>- Health policies predominantly disease oriented.<br>**Health financing**<br>- Inadequate financial resources<br>- Poorly defined health financing models<br>- Poorly performing national health insurances<br>**Supervision and accountability**<br>- Poor leadership and supervision for implementing multimorbid care guidelines.<br>- Low uptake of multimorbid care policies and programs.<br>- Poor accountability mechanisms.	**Political commitment**<br>- Support implementation of multimorbid care policies and programs.<br>- Support integrated and person-centred care reforms.<br>- Continuous review of polies and guidelines.<br>- Supporting continuous learning and improvement.
**PLATFORMS:** Coordinating services within and across sectors.	**Organisation of care**<br>- Poor care coordination among service providers<br>- Lack of standardized referral, appointment, and treatment guidelines.<br>- Poor service quality in public sector.<br>- Late attention to patients.	**Health service facilities**<br>- Decentralisation of facilities to improve access.<br>- Adequately equipped facilities<br>- Leverage health information infrastructure to improve information flow.<br>- Leverage social media platforms to intensify health promotional programs.<br>- Intensify outreach programs.
**WORKFORCE:** Re-orienting the model of care	**Personnel resources**<br>- Insufficient human resources<br>- Inadequate clinical skills for multimorbid care<br>- Poor staff-service user ratio<br>- Poor working conditions.	**Training**<br>- Promoting continuous training.<br>- Leverage key community structures from previous programs to support multimorbid care.

	- Poorly capacitated staff.<br>- Language barriers between service providers and users.	- Align service providers formal training with person-centred multimorbid care principles.<br>- Training of lower health professionals to enhance task-shifting
**TOOLS:** Equipment, medicines, and health information systems.	**Physical infrastructure and equipment**<br><br>- Infrastructure and equipment not conducive to multimorbid care.<br>- Inadequate and outdated essential equipment.<br>- Erratic supply of resources (electricity and water).<br><br>**Health information and communication systems**<br><br>- Fragmented and unreliable health information systems.<br>- Outdated and poorly managed paper-based recording systems.<br>- Poor communication systems of users' results.<br><br>**Medicines**<br><br>- Multiple prescriptions and polypharmacy cause drug burden.	**ICT technology and innovations**<br><br>- Existing ICT can be leveraged to promote m-health and m-learning of multimorbid care principles.<br><br>**Infrastructure and essential medicines**<br><br>- Develop existing infrastructure and equipment to support multimorbid care.<br>- Decentralised PHC facilities to allow easy access for users.<br>- Multi-stakeholder engagement to improve essential medicines supply chain
