# Supplementary material for: Barriers and facilitators for strengthening primary health systems for person-centred multimorbid care in low-income and middle-income countries: a scoping review
Source: BMJ Open. 2024 Nov 27;14(11):e087451. doi: 10.1136/bmjopen-2024-087451 (PMC11603689; doi:10.1136/bmjopen-2024-087451)
Supplement: Supplementary file 2 [file bmjopen-2024-087451.reviewer_comments.pdf]

PEER REVIEW HISTORY

BMJ Open publishes all reviews undertaken for accepted manuscripts. Reviewers are asked to complete a checklist review form and are provided with free text boxes to elaborate on their assessment. These free text comments are reproduced below.

ARTICLE DETAILS

Title (Provisional)

Barriers and facilitators for strengthening primary health systems for person-centred multimorbid care in Low- and Middle-Income Countries: A scoping review.

Authors

Zezai, David; van Rensburg, André Janse; Babatunde, Gbotemi Bukola; Kathree, T; Cornick, Ruth; Levitt, Naomi; Fairall, Lara R.; Petersen, I.

VERSION 1 - REVIEW

Reviewer 1

Name George, Tina

Affiliation The University of Melbourne Faculty of Medicine Dentistry and Health Sciences, Department of General practice and Primary care

Date 14-May-2024

COI None

General Comments
The paper is interesting and informative and focuses on a very relevant area. However needs some methodological clarity.<br>Though the authors were guided by the methodological framework of Arksey and O'Malley, using the JBI guidance on reporting the methodology of a scoping review may allow for more replicability of the results.<br>There are some formatting errors like missing titles to some tables and figures which needs checking.
**Specific comments**
Line 50<br>Please expand on acronyms the first time theory are used- PHC, MLTC-M,
Unsure if the methodology of a scoping review is correct as the research question is quite narrow and the authors mention that their intention is that the results of the review will influence policy and practice(Line 51- 53). This would be the purview of a systematic review where the quality of the data is also taken into consideration.<br>However if this is a scoping review it may be prudent to say that this review could identify and summarise pertinent research in this area to assist policymakers.

Please refer JBI guidance on Scoping reviews
Need to clarify some concepts like - primary health care(does it include community level interventions?), MLTC-M and what interventions are considered person centred health care systems.
In the methods section, the search strategy needs to be further clarified.<br>What were the main concepts used to construct the search strategy?<br>Were there any modifications made to translate the search strategy?<br>Were studies restricted to any language or region and why?
The background is informative but may flow better if the authors start with describing MLTC -M, its definition and its magnitude and problems specific to LMICs.<br>In the paragraph -line 13-30 – it might help to give a few examples of person centred models of care for MLTC-M that are in use in HICs to throw some light on what person centred care looks like and understand what kind of interventions the authors are referring to.
In the inclusion criteria it mentions that you looked at studies describing health systems for patient centred care. Was there a specific definition used and what sorts of interventions did it encompass? May be clearer if the inclusion criteria were in the PICOT format.
What types of studies were included?
Were only primary studies included or were secondary sources included as well like systematic reviews?
In the PRISMA diagram online supplemental fig 1 the number of articles left after duplicates were removed is not mentioned. Title screening is mentioned in the PRISMA but not in the methodology. How was this done? Was only a sample screened in duplicate?(refer Scoping review section of JBI Manual for evidence synthesis , https://jbi-global-wiki.refined.site/space/MANUAL/355862769/10.2.7+Data+extraction)
Is there a published protocol?
Online supplemental file 2 is not labelled as such in the appendix
What software was used for thematic synthesis?<br>Thematic synthesis would be useful for a systematic review and requires more in depth reporting of the methodology which is not required in as systematic review.<br>Would consider using basic qualitive content analysis. JBI manual for evidence synthesis (10.2.8 Analysis of the evidence) and video on scoping review guidance by JBI - https://youtu.be/eCyCkgBGJ1I?si=TtPPFEnf87wwPI6h
The methodology used for data analysis and synthesis needs more clarity.<br>What data extraction form was used ? Did it have extraction points regarding the framework used? For example for each paper was data extracted regarding which pillar of the health system was being described?<br>Was this inductive or deductive coding since the authors used an existing framework.

May need an example of the construction of the theme informing a category with verbatim examples to connect the conclusion of authors to the actual evidence.
Results section could also describe what interventions have been reported in the selected papers to further understand what interventions come in the category of health systems for person-centred MLTC-M care.

Reviewer **2**

Name **Askerud, Anna**

Affiliation **University of Otago, Nursing**

Date **11-Jun-2024**

COI **N/A**

This is an excellent scoping review and clearly outlines some key issues in the barriers and facilitators for strengthening care for those with multimorbidity in low- and middle-income countries. The use of evidence based methodological frameworks and health system quality frameworks really strengthens this review.

My only concerns are the multiple use of acronyms which are not explained - for example MLTC-M, CHWs, LMICs, HICs, PHC, SDG, mHealth, ICT, HCW, HIS. First mention of these need to be spelt out in full to improve readability.

Page 2 line 6 - Arksey and O'Malley's framework should be Arksey and O'Malley's methodological framework to improve understanding for the reader.

Page 4 lines 3&4 - the transition from acute to chronic disease dominance has been prevalent since the late 1990's - I'm not sure if this is true for LMIC but probably needs to be clarified.

Page 5 lines 25- 26 - I was confused with the random capital letters in HeAlth and interventioNs.

Page 5 lines 38 - 43 - The research question needs some grammar between the question and the last sentence ... at the PHC level in LMICs?'. This was formulated......

Page 7 line 17. It would be good to state that the Lancet Global health commission framework is a health system quality framework for clarity.

Page 8 line 19 - I could not see Fig 2 - the summary of key themes - this would have been a great summary and a precursor to the following discussion.

Page 19 line 25 - 5 pillars should be five pillars.

Page 21 line 3 Task - sharing should be Task sharing or The sharing of tasks with ....

VERSION 1 - AUTHOR RESPONSE

Reviewer	Comments	Response
**Reviewer 1**	The paper is interesting and informative and focuses on a very relevant area.	- *Thank you very much for your taking your time to review our work and for the positive comments.*
	However, it needs some clarity on the methodology.	- *This has been noted and adjustments have been made where necessary*
	Though the authors were guided by the methodological framework of Arksey<br>and O'Malley, using the JBI guidance on reporting the methodology of a<br>scoping review may allow for more replicability of the results.	- *The JBI guidance has been noted and applied as appropriate.*
	There are some formatting errors like missing titles to some tables and figures<br>which needs checking.	- *Several adjustments have been made in this regard.*
	Please expand on acronyms the first time theory are used- PHC, MLTC-M,	- *This has been noted and rectified.*
	Unsure if the methodology of a scoping review is correct as the research<br>question is quite narrow and the authors mention that their intention is that<br>the results of the review will influence policy and practice(Line 51- 53). This<br>would be the purview of a systematic review where the quality of the data is<br>also taken into consideration. However if this is a scoping review it may be prudent to say that this review<br>could identify and summarise pertinent research in this area to assist<br>policymakers.<br>Please refer JBI guidance on Scoping reviews.	- *This important observation has been taken note of and the view has been adopted with much thanks.*
	Need to clarify some concepts like - primary health care(does it include<br>community level interventions?), MLTC-M and what interventions are<br>considered person centred health care systems.	- *The PHC concept has been further clarified including the extend of its responsibility.*<br>*- Further, examples of healthcare interventions have been added for clarity.*

	In the methods section, the search strategy needs to be further clarified.<br>What were the main concepts used to construct the search strategy?<br>Were there any modifications made to translate the search strategy?<br>Were studies restricted to any language or region and why?	- *The strategy has been further clarified including all concepts incorporated, as well as the eligibility criteria in terms of language and setting.*
	The background is informative but may flow better if the authors start with<br>describing MLTC -M, its definition and its magnitude and problems specific to<br>LMICs.<br>In the paragraph -line 13-30 – it might help to give a few examples of person<br>centred models of care for MLTC-M that are in use in HICs to throw some light<br>on what person centred care looks like and understand what kind of<br>interventions the authors are referring to.	- *The guidance has been noted and followed and the introduction outline has been accordingly modified*<br><br>*Few additional have been made to add clarity on person-centred interventions*
	In the inclusion criteria it mentions that you looked at studies describing health<br>systems for patient centred care. Was there a specific definition used and what<br>sorts of interventions did it encompass? May be clearer if the inclusion criteria<br>were in the PICOT format.	- *This very important recommendation and the PICOT format has been adopted.*
	What types of studies were included? Were only primary studies included or were secondary sources included as well like systematic reviews?	- *A statement has been included to further clarify this.*
	In the PRISMA diagram online supplemental fig 1 the number of articles left<br>after duplicates were removed is not mentioned. Title screening is mentioned<br>in the PRISMA but not in the methodology. How was this done? Was only a<br>sample screened in duplicate?(refer Scoping review section of JBI Manual for<br><br>evidence synthesis , https://jbi-global-wiki.refined.site/space/MANUAL/355862769/10.2.7+Data+extraction)	- *This was further clarified in the text.*
	Is there a published protocol?	- *The protocol was not published.*

	Online supplemental file 2 is not labelled as such in the appendix	- *Noted and labeled as appropriate.*
	What software was used for thematic synthesis?<br>Thematic synthesis would be useful for a systematic review and requires more<br>in depth reporting of the methodology which is not required in as systematic<br>review.<br>Would consider using basic qualitive content analysis. JBI manual for evidence<br>synthesis (10.2.8 Analysis of the evidence) and video on scoping review<br>guidance by JBI - https://youtu.be/eCyCkgBGJ1I?si=TtPPFEnf87wwPI6h	- *Thank you for this, the reviewer has been guided by the well elaborate video to make the necessary adjustments.*
	The methodology used for data analysis and synthesis needs more clarity.<br>What data extraction form was used ? Did it have extraction points regarding<br>the framework used? For example for each paper was data extracted<br>regarding which pillar of the health system was being described?<br>Was this inductive or deductive coding since the authors used an existing<br>framework.<br>May need an example of the construction of the theme informing a category<br>with verbatim examples to connect the conclusion of authors to the actual<br>evidence.	- *A JBI data extraction format has been adopted. As indicated data was extracted in terms of barriers and facilitators then deductively codded according to the existing framework categories and inductively into specific subcodes for either barriers or opportunities.*
	Results section could also describe what interventions have been reported in<br>the selected papers to further understand what interventions come in the<br>category of health systems for person-centred MLTC-M care.	- *Thank you for this very important comment.*<br>- *Most of the interventions are included in the results sections in terms of how they are being promoted or inhibited by certain factors within the overall health systems framework. For instance, there is the mention of service integration, self-management*

		support, referral pathways, shared decision making, person-centered care, task shifting as examples of the existing of health systems interventions.
**Reviewer 2**	This is an excellent scoping review and clearly outlines some key issues in the barriers and facilitators for strengthening care for those with multimorbidity in low- and middle-income countries. The use of evidence based methodological frameworks and health system quality frameworks really strengthens this review.	- *Thank you very much for this positive comment and for taking your time to review our work.*
	My only concerns are the multiple use of acronyms which are not explained - for example MLTC-M, CHWs, LMICs, HICs, PHC, SDG, mHealth, ICT, HCW, HIS. First mention of these need to be spelt out in full to improve readability.	- *Have followed up and made the necessary corrections.*
	Page 2 line 6 - Arksey and O'Malley's framework should be Arksey and O'Malley's methodological framework to improve understanding for the reader.	- *Noted and added*
	Page 4 lines 3&4 - the transition from acute to chronic disease dominance has been prevalent since the late 1990's - I'm not sure if this is true for LMIC but probably needs to be clarified.	- *Clarified*
	Page 5 lines 25- 26 - I was confused with the random capital letters in HeAlth and interventioNs.	- *This was to highlight where the project name ENHANCE was derived from, which is frequently used in the text.*
	Page 5 lines 38 - 43 - The research question needs some grammar between the question and the last sentence ... at the PHC level in LMICs?'. This was formulated..	- *Noted and corrected.*
	Page 7 line 17. It would be good to state that the Lancet Global health commission framework is a health system quality framework for clarity.	*The suggestion was adopted*
	Page 19 line 25 - 5 pillars should be five pillars.	- *Corrected*
	Page 21 line 3 Task - sharing should be Task sharing or The sharing of tasks with	- *Corrected*
